# Non-Celiac Villous Atrophy—A Problem Still Underestimated

**DOI:** 10.3390/life15071098

**Published:** 2025-07-13

**Authors:** Katarzyna Napiórkowska-Baran, Paweł Treichel, Adam Wawrzeńczyk, Ewa Alska, Robert Zacniewski, Maciej Szota, Justyna Przybyszewska, Amanda Zoń, Zbigniew Bartuzi

**Affiliations:** 1 Department of Allergology, Clinical Immunology and Internal Diseases, Collegium Medicum Bydgoszcz, Nicolaus Copernicus University Torun, 85-067 Bydgoszcz, Poland; adam.wawrzenczyk@cm.umk.pl (A.W.); ewa.alska@cm.umk.pl (E.A.); robert.zacniewski@cm.umk.pl (R.Z.); maciejszota98@gmail.com (M.S.); zbartuzi@cm.umk.pl (Z.B.); 2 Student Research Club of Clinical Immunology, Department of Allergology, Clinical Immunology and Internal Diseases, Collegium Medicum Bydgoszcz, Nicolaus Copernicus University Torun, 85-067 Bydgoszcz, Poland; treichel.pawel@gmail.com; 3 Department of Nutrition and Dietetics, Collegium Medicum Bydgoszcz, Nicolaus Copernicus University Torun, 85-067 Bydgoszcz, Poland; juprz@cm.umk.pl; 4 University Hospital No. 2 in Bydgoszcz, 85-168 Bydgoszcz, Poland; amandazon6@gmail.com

**Keywords:** non-celiac villous atrophy, villous atrophy, enteropathy, immune defects

## Abstract

Non-celiac villous atrophy (NCVA) is a multifaceted and under-recognized clinical entity with an etiology beyond celiac disease. This review critically examines the diverse pathophysiological mechanisms underlying NCVA, including autoimmune enteropathies, immune deficiency-related disorders, infectious processes, drug-induced trauma, and metabolic or environmental influences. A comprehensive synthesis of peer-reviewed literature, clinical studies, and case reports was conducted, adopting a multidisciplinary perspective that integrates immunologic, infectious, metabolic, and pharmacologic insights. The literature search was performed in three phases: identification of relevant studies, critical assessment of selected publications, and synthesis of key findings. Searches were carried out in PubMed, Scopus, Web of Science, and Google Scholar databases. The final search, completed in June 2025, included international, English-language articles, electronic books, and online reports. Studies were included if they addressed NCVA in the context of pathophysiology, clinical manifestations, or management strategies, with priority given to publications from the last ten years (2015–2025). The search strategy used the primary term “*non-celiac villous atrophy*” combined with supplementary keywords such as *autoimmune enteropathy*, *common variable immunodeficiency*, *tropical sprue*, *drug-related enteropathy*, *pathophysiology*, *immunological mechanisms*, *chronic inflammation*, *genetic factors*, *environmental influences*, and *clinical management*. Histopathological evaluations reveal that NCVA often manifests with varying degrees of villous blunting, crypt hypertrophy, and intraepithelial lymphocytosis, albeit without the gliadin-specific immune response seen in celiac disease. Various immune pathways are involved, such as autoimmune deregulation and chronic inflammatory responses, while drug-induced and environmental factors further complicate its clinical picture. These findings highlight significant diagnostic challenges and underscore the need to adapt diagnostic algorithms that combine clinical history, serologic evaluations, and histopathologic analysis. In conclusion, an in-depth understanding of the heterogeneous etiology of NCVA is critical to improving diagnostic accuracy and optimizing therapeutic strategies. Future research should prioritize the identification of specific biomarkers and the development of targeted interventions to address the unique mechanisms underlying NCVA, thereby improving patient management and outcomes.

## 1. Introduction

Villus atrophy (VA) is the primary histopathological lesion found in chronic enteropathies, including both celiac disease (CD) and non-celiac enteropathies (NCEs) [1,2,3]. NCEs are a group of rare seronegative enteropathies (SNEs) with extremely diverse etiologies. The most common causes include drug-induced enteropathies, autoimmune enteropathies (AIEs), immunodeficiency-related enteropathies, infectious enteropathies, inflammatory bowel diseases, primary small bowel lymphomas and idiopathic villous atrophy (IVA) [4,5,6,7,8,9]. Meanwhile, the small intestine is the main organ responsible for the absorption of micro- and macronutrients and the first line of immune defense. For this reason, atrophy of the intestinal villi, regardless of etiology, can result in the development of dehydration, severe malnutrition and the irreversible consequences of chronic deficiencies in individual nutrients. Recognizing these complications and determining the specific cause of non-celiac villous atrophy (NCVA) is a key element in patient care [1,3,9,10]. This is particularly important in the context of recent reports indicating that the prognosis may be better for NCVAs whose cause is identifiable and successfully treated, such as drug-induced or infectious enteropathies [1]. Despite its clinical significance, NCVA remains underdiagnosed [1,3,10]. Key difficulties in diagnosis stem from the rarity of these conditions, the lack of international consensus on their nomenclature and criteria, and the overlapping clinical and histopathological manifestations, which are often confused with celiac disease. In addition, non-specific symptoms and specific challenges in differentiating from seronegative celiac disease (SNCD) complicate the diagnostic process. Also contributing to the diagnostic difficulties of this complex clinical problem of NCVA is the fact that both its incidence and clinical course are significantly modified by demographic factors such as age, gender and ethnicity [1,11,12,13,14,15,16].

Problems related to the diagnosis of NCVA impinge on the lack of standardized international guidelines on therapeutic options and monitoring of the treatment process and clinical status, including the nutritional status of patients. Moreover, NCVA often remains underdiagnosed by focusing the diagnostic process solely on celiac disease [1]. This article aims to fill this gap by comprehensively examining NCVA to improve the diagnostic approach and patient care.

The article discusses in detail the significant challenges associated with the pathogenesis and diagnosis of NCVA. Through a detailed analysis of review articles, clinical studies and case reports available in the literature, the authors of this review, taking a multidisciplinary perspective, described how non-celiac causes of villous atrophy differ in terms of pathophysiology, clinical manifestations and treatment strategies. The implications of these differences for diagnosis and management in clinical practice are assessed.

The structure of this paper follows a logical progression, beginning with an exploration of the pathophysiological mechanisms behind NCVA, including immune-mediated, inflammatory, genetic, and environmental factors. It then examines primary causes like autoimmune and immunodeficiency-related conditions, progressing to secondary and iatrogenic causes such as drug-induced and metabolic disorders. Diagnostic strategies are reviewed, emphasizing histopathology, serology, and differential diagnosis. The conclusion synthesizes these findings, highlighting their clinical implications.

This narrative review was structured in three main phases: identification of relevant literature, critical assessment of selected publications, and synthesis of key findings. To gather data, comprehensive searches were carried out in the databases PubMed, Scopus, Web of Science and Google Scholar for studies focusing on NCVA. The final search was completed in June 2025 and considered international, English-language articles, electronic books, and online reports.

The search strategy involved the primary term “non-celiac villous atrophy” combined with supplementary keywords such as autoimmune enteropathy, common variable immunodeficiency, tropical sprue, drug-related enteropathy, pathophysiology, immunological mechanisms, chronic inflammation, genetic factors, environmental influences, and clinical management. Following the initial search, duplicate records were eliminated. Abstracts of the remaining studies were reviewed to confirm their relevance. Studies were included if they explored NCVA in relation to pathogenesis, clinical features, or treatment approaches. Preference was given to publications from the past decade (2015–2025) to ensure up-to-date evidence, with earlier works included only when providing foundational insights.

## 2. Pathophysiology of Non-Celiac Villous Atrophy

**Figure 1:**
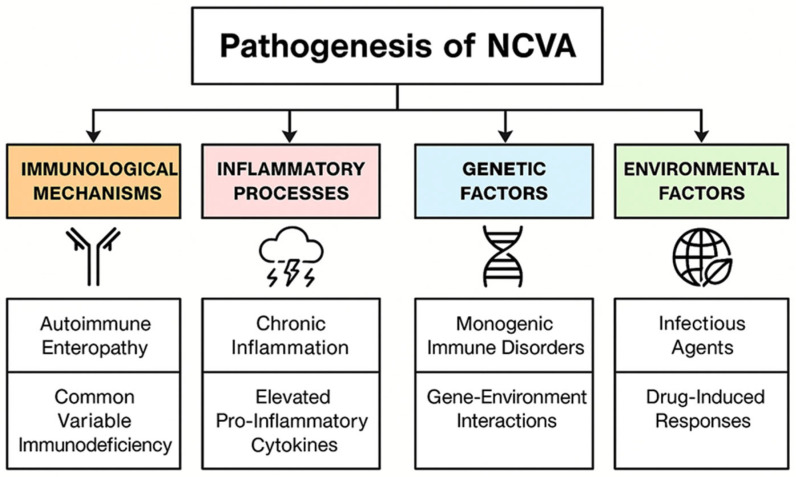
Pathogenesis of non-celiac villous atrophy [17].

Delving into the intricate mechanisms behind non-celiac villous atrophy unveils a landscape marked by diverse immunological, inflammatory, genetic, and environmental influences. The subsequent sections will explore immunological mechanisms driving conditions like autoimmune enteropathy and common variable immunodeficiency, while also examining how chronic inflammation and genetic factors contribute to the disorder. Additionally, the interplay of environmental factors, including infectious agents and drug-induced responses, further complicates the pathophysiological framework, offering insights into effective diagnostic and management strategies for this multifaceted condition. This exploration is a key foundation for understanding the leading causes and clinical manifestations [9]. The pathogenesis of non-celiac villous atrophy is shown in Figure 1.

### 2.1. Immunological Mechanisms

Immune mechanisms play a key role in developing NCVA, with different pathways underlying this pathology depending on the specific cause. Autoimmune enteropathy (AIE) is a paradigmatic example of NCVA associated with immune dysfunction, characterized by the production of autoantibodies directed against structural components of the intestines. This immune-mediated damage leads to atrophy of the intestinal villi, apoptosis of the crypts, and violation of the integrity of the mucosal barrier, contributing to chronic malabsorption and persistent diarrhea. Unlike celiac disease, in which the immune response is triggered explicitly by gliadin peptides derived from gluten, AIE is characterized by autoreactive B and T lymphocytes leading to intestinal inflammation. The clinical distinction between these conditions is crucial, as therapeutic approaches differ significantly. Histologically, AIE manifests with characteristic features such as apoptotic crypt bodies and flattened villi, underscoring the importance of combining histologic and immunologic evaluations to correctly diagnose. The systemic nature of immune dysregulation in AIE is highlighted by the prevalence of other autoimmune diseases, such as type 1 diabetes and thyroiditis. Therefore, patients with AIE benefit from a multidisciplinary approach to care that includes screening for related autoimmune diseases to provide comprehensive treatment [17].

Immunodeficiency disorders like common variable immunodeficiency (CVID) also play a significant role in the pathogenesis of NCVA. CVID is characterized by deficiencies in immunoglobulin production, resulting in weakened mucosal immunity and heightened susceptibility to chronic gastrointestinal infections. Among CVID patients, nearly 5–15% experience severe enteropathy with villous atrophy, often accompanied by clinical features such as chronic diarrhea and malabsorption. The pathophysiology of CVID-associated villous atrophy arises from chronic inflammation fueled by persistent infections and immune dysregulation. For instance, chronic norovirus infection in CVID patients amplifies inflammatory cascades and disrupts epithelial integrity, exacerbating villous damage. Targeted antiviral treatments, such as ribavirin, have shown therapeutic promise by achieving viral clearance and improving intestinal histology. This underscores the key role of chronic infections in the persistence of villi atrophy in CVID-associated cases. Moreover, immunoglobulin replacement therapy remains the gold standard treatment for CVID, reducing the incidence of infection and alleviating ongoing inflammation, helping restore intestinal health [18,19].

Another cause of the development of NCVA, related to dysregulation of the immune system, follows pharmacotherapy with preparations containing olmesartan and mycophenolate mofetil. Enteropathy secondary to olmesartan therapy results from the drug’s ability to induce T-cell activation and dysregulate cytokines, including tumor necrosis factor alpha (TNF-α) and interferon gamma (IFN-γ). These inflammatory mediators lead to epithelial apoptosis and subsequent destruction of the intestinal villi, causing symptoms such as chronic diarrhea and malabsorption that can mimic celiac disease. This overlap of clinical symptoms complicates the diagnostic process and indicates the need to include a detailed medication history. Similarly, mycophenolate mofetil disrupts the intestinal epithelium’s integrity and promotes chronic inflammation, further damaging the intestinal mucosa. It is worth noting that its enteric-coated formulation exacerbates these effects by altering absorption dynamics, increasing the likelihood of intestinal villus atrophy. Importantly, discontinuing the causative drug usually results in the resolution of symptoms and histological improvement, underscoring the need for clinicians to recognize drug-induced enteropathies and incorporate appropriate treatment quickly. Improved monitoring strategies, such as assessing cytokine profiles and T-cell activation in individuals receiving these drugs, may facilitate early detection and intervention before irreversible intestinal damage occurs [17,20].

Though NCVA may share overlapping features with celiac disease, critical distinctions allow for the differentiation of these conditions. For example, while celiac disease is strongly associated with HLA-DQ2/DQ8 haplotypes and a gliadin-specific immune response, these genetic and immunological factors are not typically involved in NCVA. This differentiation underscores the need for tailored diagnostic approaches beyond gluten-specific immune pathways. At the same time, shared mechanisms such as elevated pro-inflammatory cytokines (e.g., TNF-α and IFN-γ) complicate the clinical and histological distinction between NCVA and celiac disease. Such overlaps emphasize the importance of integrating serological, histological, and clinical data to ensure accurate diagnoses. Non-celiac conditions often arise from diverse triggers, including medications and infections, that provoke mucosal immune responses without the specificity of gluten-related disorders. Recognizing these differences is essential for devising effective patient-specific management strategies [21,22].

The persistence of chronic inflammation in NCVA, driven by deregulated cytokine profiles, is central to its pathology. Elevated levels of pro-inflammatory cytokines, such as TNF-α and IFN-γ, disrupt epithelial junctions, increasing intestinal permeability and thereby perpetuating tissue damage. These cytokines also increase T-lymphocyte activation, contributing to apoptotic cell death in the intestinal villi. This inflammatory process is particularly evident in autoimmune enteropathy and conditions such as Crohn’s disease, where the combination of cytokine storm and granulocyte activity amplifies mucosal damage. Targeted therapies that modulate cytokine activity, such as biologic drugs that inhibit TNF-α or IFN-γ, have shown potential in reducing chronic inflammation and promoting mucosal healing in patients with NCVA. This highlights the therapeutic potential of immunomodulatory approaches to address immune dysregulation in these conditions [17,19].

In conclusion, the immunological mechanisms underlying NCVA are complex and widely depend on the etiology. However, whether caused by autoimmune processes, immune deficiency infections, or drug-induced immune responses, these mechanisms underscore the need for precise diagnostic and therapeutic strategies. A deeper understanding of these immune pathways will help develop targeted interventions, improving outcomes for patients with NCVA.

### 2.2. Inflammatory Processes

Chronic inflammation represents a fundamental mechanism in the pathogenesis of NCVA, with dysregulated immune responses playing a key role in tissue damage and disease progression. Pro-inflammatory cytokines such as TNF-α and IFN-γ emerge as central players in this process, as they disrupt epithelial integrity, induce apoptosis of intestinal epithelial cells, and provoke structural damage to the small intestinal mucosa. This damage impairs the physiological intestinal barrier, manifesting in clinical symptoms such as chronic diarrhea, impaired nutrient absorption, and subsequent nutritional deficiencies. The broad impact of these inflammatory mediators on the epithelial barrier underscores their importance in the pathogenesis and clinical presentation of NCVA [17,23].

TNF-α plays a key role in perpetuating inflammatory damage in NCVA, particularly through its ability to compromise the integrity of tight junctions, thereby increasing intestinal permeability. This permeability allows antigens, toxins, and bacteria to penetrate the mucosa, triggering further immune activation and enhancing the inflammatory cycle. Additionally, the increased presence of TNF-α correlates with epithelial apoptosis, contributing to the progressive loss of villi structure and function. Targeting TNF-α with biologic therapies, such as anti-TNF agents, is an evolving strategy for alleviating chronic inflammation and restoring mucosal homeostasis in NCVA. However, the therapeutic potential of these interventions is limited by variable responses in different etiologies of NCVA, requiring more precise profiling of cytokine activity on a case-by-case basis [17,19].

IFN-γ is another pivotal cytokine involved in the pathogenesis of NCVA, particularly in autoimmune and inflammatory contexts such as autoimmune enteropathy and Crohn’s disease. IFN-γ enhances immune cell recruitment by promoting the accumulation of macrophages and cytotoxic T cells in the intestinal mucosa. This infiltration of immune cells exacerbates epithelial damage by releasing additional inflammatory mediators, creating a self-perpetuating cycle of tissue damage. Notably, systemic IFN-γ elevation is associated with more severe and refractory forms of NCVA, highlighting its role as a potential therapeutic target. However, while TNF-α-induced damage usually involves epithelial barrier disruption, IFN-γ’s contribution involves direct cytotoxicity mediated by the immune system, highlighting the need to distinguish between these pathways to optimize treatment approaches [23].

Chronic inflammation is a hallmark in the clinical picture of NCVA, which contrasts with the episodic or acute inflammatory reactions seen in other intestinal disorders. Sustained elevated levels of cytokines such as TNF-α and IFN-γ cause long-term epithelial damage and atrophy of intestinal villi, leading to persistent complications such as malabsorption and systemic nutrient deficiencies. Persistent inflammation perpetuates NCVA as a condition that requires long-term treatment strategies. Despite advances in immunomodulatory therapies, significant gaps remain in personalizing treatment to specific inflammatory profiles, leaving many patients vulnerable to the cumulative effects of chronic inflammation [17].

Crohn’s disease is a compelling example of chronic inflammation underlying NCVA, as the condition involves immune mechanisms that cause ongoing mucosal damage. The absence of celiac disease-specific markers, such as gliadin-associated antibodies or HLA-DQ2/DQ8 genetic markers, in Crohn’s disease highlights a distinct inflammatory pathway. Granulomatous inflammation, the hallmark of Crohn’s disease, contributes to the atrophy of the intestinal villi by creating strictures and transepithelial lesions that disrupt standard intestinal architecture. This pathophysiological distinction underscores the importance of distinguishing Crohn’s disease from other causes of NCVA, as its treatment typically includes immunosuppressive therapies such as corticosteroids, azathioprine, and biologic drugs. These agents directly target the inflammatory cascade, promoting mucosal healing and reducing disease progression, although their use requires careful monitoring due to potential adverse effects [17,24].

Autoimmune enteropathy and Crohn’s disease share overlapping inflammatory mechanisms in NCVA but present with distinct clinical and histological characteristics. T-cell hyperactivation and dysregulated cytokine secretion in autoimmune enteropathy result in severe mucosal inflammation, often accompanied by autoantibody production targeting intestinal components. This combination of immune responses exacerbates tissue damage and complicates treatment, as conventional therapies usually cannot control inflammation. In contrast, Crohn’s disease shows a more localized inflammatory pattern, often characterized by granulomatous changes without the widespread autoantibody activity seen in autoimmune enteropathy. This distinction is crucial for tailoring therapeutic strategies, especially since autoimmune enteropathy may require more aggressive immunosuppressive interventions [17,19].

Also, the drug-induced enteropathy described earlier is an example of the role of external factors in triggering inflammatory processes in NCVA. Recognition of drug-induced enteropathy allows for simple treatment through drug withdrawal, reinforcing the need for clinicians to incorporate detailed pharmacological assessments into their diagnostic approaches [17,20].

Infectious etiology also plays a significant role in the inflammatory damage associated with NCVA. Pathogens such as Giardia lamblia disrupt the epithelial barrier by altering tight junctions and inducing immune activation, leading to flattened villi and persistent malabsorption. The inflammatory response to infections is often associated with increased cytokine activity, further contributing to epithelial destruction. Successful treatment depends on rapid identification and targeted antimicrobial therapy. For example, metronidazole treatment of *Giardia lamblia* infection removes the cause of inflammation and facilitates histologic regeneration. This underscores the need for clinicians to include infectious agents in the differential diagnosis of NCVA to optimize patient outcomes [17,25].

Examining molecular-level inflammatory processes in NCVA provides valuable insights into its diverse etiologies. Differences in immune cell recruitment and cytokine profiles, such as the higher prevalence of T-cell-mediated inflammation in autoimmune enteropathy compared to neutrophil activity in infections, underscore the involvement of multiple pathways in the inflammatory spectrum of NCVA. For example, autoimmune enteropathy may show involvement of cytokines such as IL-17 or IL-22, while TNF-α-driven mechanisms, warranting condition-specific interventions, often characterize Crohn’s disease. Advances in the molecular understanding of these pathways may pave the way for personalized therapies, offering better disease management tailored to the unique inflammatory profiles of individual NCVA subtypes [17,23].

Future therapeutic development should prioritize targeted approaches to modulate specific inflammatory mechanisms in NCVA. The identification of novel molecular targets in cytokine pathways could revolutionize treatment, as evidenced by the partial success of existing biologics such as anti-TNF drugs. However, the heterogeneity of the inflammatory response in NCVA subtypes remains a challenge, requiring further research into the precise immune factors of villi atrophy. Integrating such findings into clinical practice will likely improve the efficacy of anti-inflammatory and immunomodulatory interventions, ultimately improving patient outcomes [17,25,26].

In conclusion, the inflammatory processes underlying NCVA are multifaceted and demand a nuanced understanding to differentiate between overlapping conditions effectively. A comprehensive approach integrating molecular insights, cytokine profiling, and clinical evaluations will enhance diagnostic accuracy and guide tailored therapeutic strategies.

### 2.3. Genetic and Environmental Factors

Genetic and environmental factors contribute significantly to the pathogenesis of NCVA. These factors act independently and synergistically to affect immune regulation, inflammatory responses, and intestinal health. Genetic predispositions, such as those in monogenic immune disorders, are fundamental to understanding NCVA. Conditions like CTLA4 haploinsufficiency and IL10RA mutations exemplify how specific genetic aberrations lead to immune dysregulation. CTLA4 haploinsufficiency is implicated in autoimmune enteropathy, primarily through dysregulated T-cell activity, culminating in sustained inflammation and tissue destruction in the small intestine. Similarly, mutations in the IL10RA gene, particularly prominent in early-onset inflammatory bowel disease (IBD), disrupt critical anti-inflammatory pathways, allowing unrestrained inflammatory responses that damage the intestinal villi. These findings underscore the importance of genetic analyses in identifying hereditary causes of NCVA, which not only aid in diagnosis but also highlight potential therapeutic targets, such as cytokine modulation or T-cell suppression, tailored to the specific genetic mechanism involved [5,27].

Regional trends in genetic mutations, such as the prevalence of IL10RA mutations in Southeast Asia, point to the need for geographically specific screening programs. In endemic regions, the burden of NCVA resulting from these mutations underscores how local epidemiological factors must guide diagnostic strategies and public health interventions. For example, infections endemic to these areas may exacerbate genetically predisposed inflammation, illustrating a gene–environment interaction that warrants proactive regional genetic and clinical monitoring. Targeted genetic testing, combined with an understanding of local environmental influences, facilitates early identification and prevention of complications in high-risk populations [27].

Genetic testing is crucial in identifying CTLA4 haploinsufficiency, which often underlies autoimmune enteropathies. Identifying inherited mutations allows early intervention through therapies such as immunomodulation, targeting specific genetic mechanisms responsible for disease progression. Moreover, the findings argue for family screening, since relatives of affected individuals may share genetic susceptibilities. Such a comprehensive approach—including genetic diagnosis and family risk assessment—improves both individual and population management of NCVA, reducing delays in diagnosis and associated morbidity [17].

Environmental factors have a significant impact on the etiology and progression of NCVA. Infection, dietary exposure, and poor sanitation are key factors, often working in tandem with genetic predisposition. Tropical sprue is an illustrative example, as it is endemic in regions with inadequate sanitation. Chronic bacterial infections associated with tropical sprue cause persistent mucosal inflammation, disrupt epithelial integrity, and cause atrophy of intestinal villi. Improvements in public health infrastructure, particularly sanitation, could mitigate these environmental factors and significantly reduce the incidence of NCVA in these vulnerable populations [25].

Environmental factors extend beyond infections to include dietary deficiencies and contaminated food or water sources, which often compound the damage caused by infections. For example, tropical sprue is exacerbated by nutritional deficiencies, particularly in folate and vitamin B12. Correcting such deficiencies through dietary supplementation, alongside antimicrobial therapy, forms an essential component of treatment. This multifaceted approach underscores the necessity of integrating nutritional support into the management strategies for environmentally mediated NCVA [17,25].

Interactions between genetic predisposition and environmental factors provide a complex framework for the pathogenesis of NCVA. Tropical sprue is an example of this interaction, in which genetic susceptibility interacts with chronic exposure to bacteria to disrupt gut microbiota and epithelial function. Understanding the dynamics of these gene–environment interactions is critical to developing effective prevention and treatment strategies. Future studies focusing on this interaction may provide practical information on mitigating intestinal damage in genetically predisposed individuals with coexisting increased exposure to environmental factors [5].

Epigenetic mechanisms provide another layer of complexity, as environmental factors may modulate gene expression patterns linked to NCVA. For instance, changes in Wnt pathway gene expressions, such as altered DVL2 and CCND2 levels observed in pediatric Marsh 3b celiac patients, suggest that environmental stressors could exacerbate genetic vulnerabilities. Epigenetic modulation, potentially induced by infections and/or nutritional deficiencies, may influence disease severity or progression, underscoring the importance of addressing modifiable environmental factors in high-risk populations [28].

The integration of genetic mapping and environmental risk profiling holds promise for improving NCVA risk prediction models. Determining how environmental exposures, such as bacterial overgrowth or parasitic infections, interact with genetic mutations can inform prevention strategies and optimize therapeutic management. For example, practical insights from such studies may enable clinicians to intervene early in patients with known genetic mutations exposed to high-risk environments, reducing the long-term burden of villous atrophy [17].

Pharmacogenomics, which studies how genetic factors influence responses to drugs, is particularly relevant in cases of drug-induced NCVA. Some drugs, such as olmesartan and mycophenolate mofetil, induce immune reactions leading to villous atrophy, especially in those with genetic susceptibility. A more thorough understanding of pharmacogenomic interactions in these cases could enhance drug safety by identifying at-risk patients or suggesting alternative treatments. Preventive screening using pharmacogenomic markers, combined with close monitoring of drug effects, could significantly reduce the number of NCVA cases associated with drug therapies [20].

The role of medications in triggering NCVA underscores the necessity of tailoring treatment plans to individual genetic and environmental profiles. For example, in transplant patients developing villous atrophy due to immunosuppressive drugs, carefully tapering these medications while maintaining graft function could prevent irreversible intestinal damage. Such individualized approaches illustrate the importance of integrating pharmacological history into the overall diagnostic and therapeutic framework for NCVA [25].

Multifactorial contributions of genetic and environmental factors render NCVA a complex pathological entity necessitating an integrated approach to care. For instance, in CVID, genetic susceptibility to immune dysfunction interacts with recurrent infections, resulting in villous atrophy. Management strategies combining immunoglobulin replacement for CVID-related immune deficits and targeted treatments for recurrent infections demonstrate how addressing genetic and environmental dimensions can optimize patient outcomes [8,27].

Tailored therapies addressing specific genetic predispositions and environmental triggers exemplify effective management of NCVA. Whether genetic interventions, such as immunoglobulin transfusions in CVID, or environmental strategies, such as controlling parasitic infections, these approaches underscore the need for personalized care. Multidisciplinary collaboration is key to addressing the multifaceted nature of NCVA. Integrating the expertise of gastroenterologists, immunologists, and geneticists facilitates a comprehensive approach, ensuring that all factors are considered when making diagnostic and therapeutic decisions [8,17].

Future research must prioritize exploring genetic and environmental interactions, paying particular attention to underrepresented populations and regional differences. The higher prevalence of specific mutations, such as IL10RA in Southeast Asia and endemic infections, such as tropical infections, underscores the need for geographically tailored diagnostic and management strategies. Investigating these factors globally could significantly improve the understanding of NCVA pathogenesis and enhance patient care worldwide [27].

The genetic and environmental factors influencing NCVA form a complex, multifactorial network. A better understanding of these interactions through integrative research will pave the way for personalized, effective therapeutic strategies to reduce the burden of this challenging condition.

## 3. Primary Causes and Clinical Manifestations

Delving into the complex clinical picture of non-celiac intestinal villous atrophy, this section delves into its major causes and clinical manifestations, highlighting the role of various autoimmune conditions, immunodeficiency disorders, and infectious etiologies. Understanding these underlying factors will give readers insights into this multifaceted gastrointestinal disorder’s diverse presentations and complexities. The subsequent discussions will outline the key pathological mechanisms and clinical implications that arise from each etiology, setting the stage for a deeper understanding of non-celiac villous atrophy’s impact on patient health. Although villous atrophy is classically associated with celiac disease, a wide spectrum of non-celiac etiologies must be considered. Table 1 outlines the most common causes of NCVA along with their distinguishing characteristics.

### 3.1. Autoimmune Conditions

The complexities of autoimmune conditions reveal their profound impact on non-celiac villous atrophy, underscoring the intricate relationship between systemic immune dysregulation and gastrointestinal health. This section will explore key disorders such as autoimmune enteropathy, highlighting their distinctive clinical and histological features and associated autoimmune diseases that frequently coexist. By examining the underlying mechanisms and therapeutic approaches, readers will gain insight into the broader implications of autoimmunity on gut function and patient management within the multifaceted clinical course of these gastrointestinal disorders.

#### 3.1.1. Autoimmune Enteropathy

Autoimmune enteropathy (AIE) represents a rare but significant pathogenetic factor in intestinal villous atrophy, characterized by specific clinical and histological features that distinguish it from other intestinal pathologies. Characteristic manifestations of AIE include persistent diarrhea, malabsorption, and weight loss, which often coexist with abnormal histopathological findings, such as marked villous atrophy, minimal intraepithelial lymphocytosis, increased numbers of apoptotic bodies in crypts, and a significant absence of cup cells or Paneth cells [29]. These histological distinctions are critical in accurately diagnosing AIE, particularly in differentiating it from celiac disease. The lack of goblet cells, a feature unique to AIE, underscores the extensive mucosal damage and points to an underlying autoimmune mechanism not observed in celiac disease. Similarly, the minimal intraepithelial lymphocytosis contrasts sharply with the prominent lymphocytic infiltration characteristic of celiac disease, reflecting fundamental differences in immune pathophysiology. The increase in crypt apoptotic bodies highlights the severe epithelial injury driven by immune dysregulation, offering insight into the mechanisms underlying villous atrophy in AIE patients. These findings emphasize the importance of histological analysis in establishing a definitive diagnosis, enabling the effective differentiation of AIE from other causes of villous atrophy.

Diagnostic criteria for AIE have evolved significantly, moving away from relying on serological markers to emphasize objective histologic features and exclude other potential causes of villous atrophy. Initially, antibodies to enterocytes were a key diagnostic criterion, but these antibodies are detected in only about 50% of AIE cases, limiting their reliability and sensitivity [29,30]. The updated diagnostic approach emphasizes the importance of histologic findings, such as crypt apoptosis and villous atrophy, as more consistent markers, while emphasizing the exclusion of other etiologies, such as infectious or drug-induced causes. This shift in diagnostic focus underscores the need for an integrative approach that combines histopathological evaluation with clinical presentation. For example, the persistence of chronic diarrhea and malabsorption that fails to respond to dietary modification provides essential context to confirm an AIE diagnosis. Improved diagnostic guidelines ultimately facilitate more accurate identification of AIE and allow for the introduction of appropriate treatment strategies.

AIE’s strong association with other autoimmune conditions further underscores its systemic nature and complexities. Concomitant autoimmune disorders are seen in up to 70% of AIE cases, with conditions such as type 1 diabetes, thyroid disorders, and systemic autoimmune diseases among the most common comorbidities with AIE [17,30]. This high prevalence of associated autoimmune diseases suggests a shared predisposition rooted in immune dysregulation, often involving overlapping genetic and immunological pathways. For instance, type 1 diabetes mellitus, commonly observed in AIE patients, shares susceptibility loci such as CTLA4 and PTPN22, which mediate immune responses and contribute to systemic autoimmunity. Similarly, thyroid disorders, particularly Hashimoto’s thyroiditis, further illustrate the interplay between gastrointestinal and systemic autoimmune processes. Recognizing these associations is crucial for clinicians, as identifying concurrent autoimmune diseases allows for a comprehensive treatment strategy that addresses the broader immune dysfunction present in AIE patients. Screening for comorbid autoimmune diseases not only aids in early detection but also ensures that systemic manifestations are promptly addressed, enhancing overall patient outcomes [29,30,31].

Genetic investigations have illuminated critical pathogenic mechanisms underlying AIE, with identified mutations such as CTLA4 haploinsufficiency and nuclear factor kappa B subunit 1 mutations shedding light on the disorder’s immune dysregulation [19]. CTLA4 haploinsufficiency, a T-cell regulation defect, exemplifies the role of genetic predispositions in perpetuating chronic inflammation and intestinal damage. This mutation disrupts immune homeostasis, leading to heightened T-cell activation and sustained mucosal injury. Similarly, nuclear factor kappa B subunit 1 mutations highlight the role of transcriptional dysregulation in driving chronic inflammation. These findings have practical implications, as they pave the way for precision medicine approaches tailored to a patient’s genetic profile. For example, targeted genetic counseling and interventions can address the specific mechanisms contributing to immune dysregulation, offering personalized treatment options. Further research into the genetic basis of AIE deepens understanding of its pathogenesis and opens avenues for novel therapeutic strategies.

Immunosuppressive therapy remains the gold standard treatment for AIE, with glucocorticoids serving as the primary intervention due to their rapid efficacy in relieving inflammation and restoring mucosal integrity. The use of glucocorticoids provides significant symptomatic relief and facilitates the restoration of intestinal architecture in most patients [25,30]. However, the chronic nature of AIE requires long-term treatment, often through the inclusion of steroid-sparing agents, such as azathioprine and mycophenolate mofetil, to minimize glucocorticosteroid-related side effects. Significant advances in AIE therapy have been made with the introduction of biologic drugs such as infliximab (anti-TNF-α) and vedolizumab (anti-integrin). Biologic therapy has shown particular promise in refractory cases unresponsive to conventional immunosuppressive therapies. Biologic drugs target specific cytokine pathways, representing a more precise approach to treating severe inflammation. Personalizing treatment regimens based on individual patient responses to selected therapies underscores the importance of patient-centered care in treating AIE. Despite these advances, AIE remains a complex condition to treat, requiring continuous monitoring and adjustment of the therapeutic process to maintain complete disease control and minimize relapses.

The chronicity of AIE presents significant management challenges, particularly concerning its long-term complications and high relapse rates. Patients often experience persistent malabsorption and secondary nutrient deficiencies due to incomplete mucosal recovery, necessitating routine nutritional assessments and supplementation. Iron, calcium, and fat-soluble vitamins are prone to depletion, highlighting the need for tailored nutritional support to manage these deficiencies. In severe cases of refractory AIE where conventional therapies fail to achieve mucosal healing, parenteral nutrition may become necessary to provide essential nutrients and support recovery [17,32].

Regular follow-up with periodic endoscopic evaluations is critical for detecting complications such as refractory enteropathy. Adjusting therapeutic regimens based on disease progression underscores the importance of sustained, multidisciplinary care in managing AIE. As treatment options evolve, integrating novel therapies such as biologics with traditional management approaches offers hope for improved outcomes in this complex condition. In conclusion, the multifaceted nature of AIE necessitates a comprehensive approach to diagnosis and management, emphasizing the integration of histological, clinical, and therapeutic strategies [29,31].

#### 3.1.2. Associated Autoimmune Disorders

Autoimmune disorders represent a significant category among the causes of villous atrophy, driven by shared immunopathogenic mechanisms involving chronic inflammation, dysregulated immune responses, and the production of autoantibodies. Commonly associated autoimmune conditions such as type 1 diabetes mellitus (T1DM), autoimmune thyroiditis, systemic lupus erythematosus (SLE), and autoimmune polyglandular syndromes (APS) further illustrate the systemic nature of these disorders, with intestinal involvement often adding complexity to their clinical presentation. Understanding the interplay between systemic and localized autoimmune activity is critical to advancing diagnostic precision and targeted therapeutic strategies in cases of villous atrophy [17,25].

Chronic inflammation accompanying autoimmune diseases is central to the pathogenesis of intestinal villous atrophy. During T1DM or autoimmune thyroiditis, an inflammatory response disrupts immune regulation and causes systemic and tissue-specific damage, including damage to the intestinal epithelium. Cellular mediators such as helper T cells and cytokines such as TNF-α and interferon-gamma (IFN-γ) are specifically involved in this inflammatory cascade. The resulting immune dysregulation exacerbates tissue damage and contributes to sustaining the inflammatory response, underscoring the cyclical nature of these processes. Studying the cross-reactivity of the immune response in autoimmune conditions may provide insight into the mechanisms of intestinal damage and help identify biomarkers of villous atrophy in such cases [29].

Autoantibody production is another hallmark of autoimmune disorders that critically influences the development of villous atrophy. Antibodies targeting enterocytes may lead to epithelial damage and disrupt the intestinal mucosal barrier. For instance, in systemic autoimmune disorders, these autoantibodies amplify immune-mediated tissue injury, complicating the diagnosis and management of coexistent villous atrophy. The presence of anti-enterocyte antibodies in patients with autoimmune conditions highlights an avenue for future research into their diagnostic value and potential as therapeutic targets. Refining serological tools to detect such antibodies more consistently could enhance diagnostic accuracy and offer earlier intervention opportunities [29].

The gastrointestinal consequences of autoimmune disorders, including persistent diarrhea and nutrient malabsorption, underscore the need to thoroughly screen individuals with systemic conditions for signs of intestinal damage. For example, malabsorption in T1DM patients may lead to glycemic instability and suboptimal management of diabetes, particularly if villous atrophy remains undiagnosed. Early detection of gastrointestinal lesions is essential to prevent exacerbation of systemic disease and related complications. Comprehensive screening protocols combining serologic, histologic, and clinical evaluations could improve detection rates and provide rapid intervention in these patients [17].

Type 1 diabetes mellitus demonstrates robust associations with villous atrophy, reflecting a complex interplay of immune responses and genetic predispositions. Shared genetic loci, such as HLA-DQ2 and HLA-DQ8, offer a partial explanation for the frequent coexistence of these two conditions. These loci mediate immune responses that target pancreatic β-cells and intestinal epithelial cells, resulting in dual organ involvement [3]. This relationship is clinically relevant because undiagnosed intestinal villous atrophy in patients with T1DM often leads to poorer glycemic control due to impaired absorption of carbohydrates, proteins, and fats. Although gluten-free diets have shown promise in improving intestinal health and glycemic stability in patients with T1DM, further research is warranted to determine long-term efficacy and optimize dietary recommendations for this particular subgroup of patients [17].

Autoimmune thyroiditis, encompassing disorders like Hashimoto’s thyroiditis and Graves’ disease, is frequently observed in individuals with villous atrophy and demonstrates the broader systemic impact of immune dysregulation. Genetic studies reveal shared susceptibility loci, such as CTLA4, which regulate immune responses and contribute to the overlapping pathologies of thyroid dysfunction and intestinal inflammation. The disruption of gut epithelial integrity in thyroiditis may arise from cytokine-mediated inflammatory pathways, with elevated levels of IFN-γ and TNF-α linked to villous damage. Thyroid dysfunction further exacerbates malabsorption-associated complications, such as osteoporosis and anemia, underscoring the need for an integrated approach to managing coexisting thyroid and gastrointestinal disorders. Individualized therapeutic strategies based on the synergistic effects of thyroid hormone replacement and appropriate nutritional intervention are essential to improve patient outcomes, including normalization of systemic metabolism and gut health [25,29].

Systemic lupus erythematosus, though less commonly linked to villous atrophy, underscores the broader implications of systemic autoimmune activity on gastrointestinal function. The intestinal damage associated with SLE is often mediated by antibodies to enterocytes, which impair epithelial barrier function and contribute to chronic inflammation. Elevated levels of inflammatory cytokines, such as IL-6 and TNF-α, observed in SLE further suggest a role for systemic immune dysregulation in intestinal damage. Clinical manifestations, including diarrhea and malabsorption, often mimic or overlap with other causes of intestinal villous atrophy, complicating the diagnostic process. This complexity underscores the importance of distinct serologic and histologic markers to distinguish villi atrophy associated with SLE from other intestinal diseases. Treatment strategies incorporating corticosteroids and biologics like rituximab target systemic immune activity and have shown promise in restoring intestinal function and mitigating symptoms [17].

Autoimmune polyglandular syndromes represent a particularly complex disease process in which atrophy of the intestinal villi is observed, following the coexistence of multiple autoimmune diseases, which contribute to amplifying systemic immune dysfunction. APS type II, characterized by the simultaneous presence of adrenal insufficiency, T1DM, and autoimmune thyroiditis, highlights how overlapping conditions can predispose individuals to intestinal inflammation and villi damage. Genetic predispositions, particularly shared HLA haplotypes and immune checkpoint variants, underlie these patients’ need for genetic screening. The combined effects of systemic inflammation, nutrient malabsorption, and associated endocrine dysfunction necessitate individualized treatment approaches. Hormone replacement therapies, such as those addressing cortisol deficiency, immunosuppressive agents, and nutritional support, are critical to managing the diverse manifestations of APS [33].

Understanding the interconnected mechanisms driving autoimmune-related villous atrophy is essential for advancing diagnostic and therapeutic strategies. The co-occurrence of autoimmune conditions requires a comprehensive approach combining genetic, serological, and histological evaluations to address the root causes of intestinal damage. Collaborative care involving endocrinologists, immunologists, and gastroenterologists is key to managing the complex interaction of systemic and gastrointestinal symptoms in affected patients. Long-term monitoring of autoimmune patients with intestinal villous atrophy is essential to assess treatment efficacy and prevent complications such as persistent malabsorption and secondary nutritional deficiency. Identifying standard immune mechanisms symptomatic of autoimmune disorders should ultimately improve the diagnostic process and therapeutic outcomes associated with improvements in the patient’s clinical condition, including intestinal health.

### 3.2. Immunodeficiency Disorders

Immunodeficiency disorders are an essential category of causes of intestinal villous atrophy, mainly through mechanisms that weaken immune defenses and promote chronic intestinal inflammation. Common variable immunodeficiency is an example of the significant impact of an impaired immune response on gastrointestinal health. Literature data show that approximately 5–15% of CVID patients develop severe enteropathy manifested by persistent diarrhea, weight loss, and malabsorption syndrome [8,18,34]. These symptoms underscore the systemic consequences of immune deficiency and its role in exacerbating intestinal damage. The interplay between systemic immune dysfunction and localized inflammation in CVID reveals the necessity of targeted therapeutic strategies that address infection control and immune restoration.

The pathophysiology of villous atrophy in CVID is due to insufficient production of immunoglobulins (IgG, IgA, and IgM), which play a key role in maintaining intestinal barrier function and eliminating pathogens. The body’s inability to produce adequate antibodies results in recurrent, persistent infections that perpetuate the cycle of inflammation and mucosal damage, leading to chronic villous atrophy [18]. This chronic inflammation sustained by repeated infections points to the importance of immunoglobulin replacement therapy to restore immune function and alleviate gastrointestinal complications [29]. Understanding the underlying immune mechanisms in CVID-associated villous atrophy can enhance therapeutic approaches that combine replacement therapy with interventions targeting infections and chronic inflammation.

The histological presentation of villous atrophy in CVID often lacks specificity, complicating its differentiation from other NCVA causes. The absence of distinct histopathological markers necessitates reliance on clinical history, including recurrent infections, and laboratory findings, such as decreased immunoglobulin levels, for accurate diagnosis [17]. This diagnostic complexity underscores the importance of integrating clinical and laboratory data to establish a precise diagnosis. Additionally, improved histological and molecular techniques may further refine the identification of CVID-related NCVA among overlapping intestinal pathologies. Moreover, it has been noted that inflammatory factors associated with CVID contribute significantly to the development of seronegative villous atrophy (SNVA), a group of conditions widely classified as celiac or non-celiac related [25]. The inflammatory nature of CVID is closely related to the immunological contribution to the pathophysiology of NCVA, showing overlapping mechanisms along with other causes such as graft-versus-host disease and Crohn’s disease [25].

Management strategies for CVID highlight the efficacy of immunoglobulin replacement therapy in reducing infection frequency and alleviating gastrointestinal inflammation, which may lead to partial or complete resolution of villous atrophy [34]. The effect of immunoglobulin therapy on the ability to regenerate intestinal villi underscores its crucial importance in combating the root causes of NCVA in CVID patients. However, the literature shows that the response to treatment varies, requiring personalized care plans that consider the degree of mucosal damage and the patient’s overall immune status.

Targeted antiviral therapies, such as ribavirin for chronic norovirus infections, have demonstrated significant benefits in clinical outcomes for CVID patients, particularly in resolving diarrhea and facilitating histological recovery of the villous structure [18]. These findings highlight the need for precise identification and aggressive management of specific infections contributing to villous atrophy in CVID. However, the efficacy of such targeted approaches also depends on more comprehensive strategies that concurrently address immune dysfunction and mucosal damage.

Chronic infections, such as those caused by noroviruses, are particularly harmful in CVID because of a significantly impaired immune response. Norovirus infections in CVID patients often persist for long periods, sometimes more than 9–12 months, leading to severe diarrhea and long-term intestinal damage [18]. This illustrates the critical need to detect infection early to rapidly implement innovative antiviral therapies, thus preventing the onset of long-term gastrointestinal complications. Increased research into the mechanisms underlying chronic infections in CVID may facilitate the development of new antiviral agents capable of achieving sustained eradication of the virus.

Clinical case studies involving ribavirin treatment for norovirus infections in CVID patients have demonstrated marked clinical improvement, including resolution of diarrhea and histological recovery of villous atrophy [18]. These results suggest that when appropriately implemented, targeted therapies can significantly improve patient outcomes. However, variability in individual responses to ribavirin emphasizes the need for ongoing research to determine optimal treatment protocols and to explore alternative antiviral agents that may offer broader efficacy.

The relationship between chronic infection, immune dysregulation, and intestinal inflammation in CVID highlights the complexity of managing NCVA in this population. Persistent infections provoke mucosal damage and perpetuate inflammatory responses that sustain villous atrophy. Successful management strategies must therefore integrate pathogen-specific therapies with interventions aimed at modulating inflammation and restoring mucosal integrity [17]. Continued exploration of the interplay between immune dysfunction and chronic inflammation is crucial to advancing treatment paradigms in CVID-associated NCVA.

HIV/AIDS represents another immunodeficiency disorder contributing to NCVA, with multifactorial mechanisms that include enterocyte injury, partial villous atrophy, and crypt hyperplasia. These structural changes disrupt nutrient absorption and result in chronic diarrhea and malabsorption, further exacerbating patient morbidity. The pathogenesis of malabsorption in HIV is compounded by pathogens such as Cryptosporidium, which intensify epithelial damage and lead to excessive protein and fat losses, compounding the systemic consequences of villous atrophy [35]. These findings highlight the need for integrated therapeutic interventions that address both the infectious and structural components of HIV-associated NCVA. Additionally, the association of HIV enteropathy with inflammatory and immune-mediated forms of villous atrophy reflects its inclusion within the broader spectrum of conditions like SNVA [25].

The metabolic consequences of malabsorption in HIV infections are profound and include complications such as osteomalacia, anemia, and hypocholesterolemia due to impaired nutrient absorption. These systemic effects underscore the need for comprehensive management strategies that combine antiviral therapies with nutritional interventions to improve overall health outcomes. Although oral half-elemental diets and parenteral nutrition have shown promise in stabilizing patients, their success depends on addressing the underlying intestinal damage caused by HIV in the therapeutic process [35]. This underscores the importance of a multidisciplinary approach to managing malabsorption in HIV patients with villous atrophy.

Immunoglobulin replacement therapy remains the gold standard treatment for immunodeficiency-associated NCVA, with particular efficacy in reducing the incidence of infection and inflammation in CVID patients, thereby improving gastrointestinal health [34]. Additional therapies targeting specific infections, such as ribavirin for norovirus in CVID, have proven essential in mitigating villous atrophy and reducing gastrointestinal morbidity [18]. These therapeutic advancements underscore the importance of tailoring treatment approaches to the underlying immune dysfunction and etiology of NCVA.

Addressing persistent malabsorption through nutritional support, including parenteral nutrition, remains vital in preventing secondary deficiencies and improving patient outcomes [34]. This highlights the significance of integrating dietary and nutritional strategies into the broader management plan for immunodeficiency-related NCVA. Moreover, the use of immunosuppressive agents and steroids in refractory cases emphasizes the need for individualized treatment protocols that account for disease severity and underlying immunological factors [17].

The diagnostic challenges of immunodeficiency-associated NCVA stem from overlapping clinical and histological features with other causes of NCVA. Integrating clinical history, particularly recurrent infections, with immunoglobulin profiling is critical for accurate diagnosis. Advances in genetic testing, which have identified monogenic mutations such as CTLA4 haploinsufficiency, among others, offer new diagnostic and therapeutic opportunities by elucidating the mechanisms of immune dysregulation contributing to villi atrophy [17]. In addition, incorporating stool analysis and pathogen identification into diagnostic protocols is essential to distinguish immune-mediated NCVA from other etiologies and implement targeted therapeutic strategies [35]. Moreover, recognizing the spectrum of infections, such as bacterial overgrowth and HIV enteropathy, which have been identified as causes of SNVA, strengthens the diagnostic process [25].

In conclusion, the complex interplay of immune dysfunction, chronic infections, and persistent inflammation in immunodeficiency-associated NCVA necessitates an integrated diagnostic and therapeutic approach. Tailoring management strategies to address systemic immune deficits and localized gastrointestinal damage is essential for improving outcomes in this vulnerable population.

### 3.3. Infectious Etiology

Infectious etiologies play a central role in the development of NCVA and encompass a diverse array of parasitic, viral, bacterial, and region-specific infections that induce damage to the intestinal villi. Parasitic infections, such as *Giardia lamblia* and *Cryptosporidium,* are prominent contributors, each utilizing distinct mechanisms to disrupt intestinal integrity. *Giardia lamblia* primarily causes mucosal inflammation and increases epithelial permeability, leading to nutrient malabsorption, diarrhea, and subsequent weight loss and nutritional deficiencies [25,36]. The disruption of tight junctions in epithelial cells caused by *Giardia* further exacerbates malabsorption. On the other hand, *Cryptosporidium* acts through intracellular parasitism, inducing epithelial cell death and flattening of the villi, which manifests as more severe malabsorption. The contrasting pathogenetic mechanisms between these parasites indicate their different adaptive strategies in the intestinal environment. This dichotomy also underscores the need for precise diagnostic tools to differentiate between parasitic infections, as their treatment strategies differ significantly.

Treating parasitic infections is critical in reversing the damage associated with intestinal villous atrophy. Medications such as metronidazole, which targets *Giardia lamblia* through its anaerobic energy metabolism, and nitazoxanide, effective against *Cryptosporidium*, have demonstrated clinical efficacy in resolving symptoms and structural abnormalities within the intestinal villi [25,36]. Post-treatment biopsies often confirm recovery of villous structures, indicating the reversible nature of parasite-induced NCVA when managed effectively. However, treatment resistance or recurrence cases, potentially linked to poor sanitation and reinfection, highlight the critical need for comprehensive public health strategies in endemic regions. This emphasizes that, beyond pharmacological interventions, addressing environmental risk factors is essential to achieving sustained resolution.

Persistent parasitic infections are also associated with systemic immune dysregulation, further compounding villous atrophy. Long-term infections trigger inflammatory responses characterized by activating a cascade of cytokines, including TNF-α, which perpetuate epithelial cell apoptosis and sustain mucosal damage [17]. Despite initial therapeutic success, this immune-mediated component highlights why some patients experience recurrent or refractory villous atrophy. Understanding these interactions between parasitic infections and immune responses offers potential for adjunctive therapies that modulate inflammation, providing a more holistic approach to treatment and preventing relapse.

Viral infections, particularly noroviruses, are a critical pathogenetic factor in NCVA, especially in immunocompromised individuals. Norovirus infections are particularly problematic in conditions such as CVID, in which chronic viral infection leads to treatment-resistant diarrhea and permanent damage to intestinal villi. The mechanisms by which noroviruses disrupt intestinal homeostasis include prolonged viral replication, which impedes epithelial regeneration and impairs nutrient absorption. In contrast to parasitic infections, the prolonged evasion of the immune system seen in chronic norovirus infections prolongs recovery time and makes conventional symptomatic treatment inadequate. This calls for targeted antiviral therapies, such as ribavirin, which has demonstrated promising outcomes in reducing viral loads and facilitating villous recovery in CVID patients [18]. These therapies underscore the importance of individualized medicine, particularly in cases where persistent infections extend the timeline of gastrointestinal damage.

In addition to antiviral treatments, early diagnostic measures are essential in mitigating the impact of viral infections on intestinal health. Advanced diagnostic tools, such as stool and polymerase chain reaction (PCR) testing for norovirus and other viral pathogens, enable earlier identification and allow timely initiation of targeted therapies [17]. Addressing viral infections promptly can minimize the potential for irreversible villous damage and prolonged malnutrition. This highlights the critical need for proactive diagnostic measures in immunocompromised populations, where undetected chronic viral infections can lead to compounded morbidity.

Bacterial infections represent another essential category of causes of NCVA, with pathogens such as *Mycobacterium tuberculosis* and *Tropheryma whipplei* exhibiting unique mechanisms for inducing intestinal damage. *Mycobacterium tuberculosis* triggers a granulomatous inflammatory response in intestinal tuberculosis, leading to villous destruction and impaired absorption [17,25]. On the other hand, Whipple’s disease, caused by *Tropheryma whipplei*, infiltrates the lamina propria, causing macrophage accumulation and characteristic flattening of the villi. These pathophysiological mechanisms underscore the importance of recognizing bacterial infections as distinct factors contributing to NCVA, each requiring a specific diagnostic and therapeutic approach.

Addressing bacterial infections involves prolonged antimicrobial therapy to eradicate the causative pathogens and foster intestinal recovery. For instance, intestinal tuberculosis typically requires combination therapy with isoniazid, rifampin, and ethambutol, while Whipple’s disease responds well to ceftriaxone followed by long-term maintenance therapy using trimethoprim–sulfamethoxazole [17]. The prolonged treatment durations reflect the chronic nature of bacterial-induced villous atrophy and emphasize the importance of compliance and follow-up in achieving successful outcomes. Incorporating molecular techniques, such as tissue PCR for *Tropheryma whipplei* or acid-fast staining for *Mycobacterium tuberculosis*, into diagnostic protocols is essential in confirming bacterial etiologies rapidly and initiating effective treatment [25]. The increased diagnostic sensitivity ultimately aims to prevent the progression of villous atrophy caused by delayed diagnosis or inappropriate therapeutic management.

Tropical splenomegaly syndrome is a geographically specific infectious etiology associated with NCVA in tropical regions, characterized by bacterial dysbiosis and nutritional deficiencies. This multifactorial condition manifests through chronic diarrhea, weight loss, and malabsorption, partly driven by bacterial toxins inducing mucosal inflammation [25]. Treating tropical sprue typically involves long-term antibiotic therapy (usually tetracycline), supplemented by folate to address associated micronutrient deficiencies. This combination has effectively controlled symptoms and promoted the regeneration of villous structures [25,37]. However, the condition underscores the broader impact of environmental factors on gastrointestinal health, pointing to the pressing need for public health interventions to improve hygiene and reduce disease prevalence in affected regions [17].

In immunocompromised populations like solid organ transplant (SOT) recipients, infectious NCVA is often linked to opportunistic pathogens that exploit the patient’s weakened immunity. Parasitic infections, such as *Giardia lamblia*, are particularly severe in these individuals and manifest as persistent diarrhea and extensive damage to intestinal villi compounded by the immunosuppressive therapies required after transplantation. Effective management of such cases includes simultaneous infection control and adjustment of immunosuppressive drugs. For example, reducing doses of azathioprine with concomitant administration of antiparasitic medications such as metronidazole has proven effective in restoring intestinal integrity and resolving symptoms [36]. These cases underscore the special care required in treating SOT recipients, where both immunosuppression and treatment of infection must be carefully calibrated to improve outcomes.

Preventive measures, including routine screening for infections in vulnerable populations, hold significant promise in addressing NCVA. Prophylactic antimicrobial strategies and routine monitoring for gastrointestinal infections can identify potential triggers before they lead to severe villous damage [17]. These strategies reflect the need for an integrative care model that combines proactive patient monitoring with individualized treatment plans to reduce the burden of infectious NCVA across diverse populations. Infectious etiologies thus represent a challenging yet pivotal area in the understanding and managing of NCVA, with significant opportunities for further research and clinical advancements.

## 4. Secondary and Iatrogenic Causes

Research available in the current scientific literature on the clinic of NCVA indicates that autoimmune and/or infectious processes are not the only factors involved in the pathogenesis but are also significantly influenced by secondary and iatrogenic factors. Through understanding the role of drug-induced enteropathy, environmental influences, and metabolic disorders, a more comprehensive picture emerges of how these external elements complicate the described disease process. This section discusses the intricacies of the implications of drug-induced complications. It highlights the importance of recognizing these factors in the overall diagnostic and therapeutic approach to treating villous atrophy. By integrating this knowledge, clinicians can develop strategies to mitigate risks and optimize patient outcomes.

### 4.1. Drug-Induced Enteropathy

Drug-induced enteropathy is an essential cause of non-celiac villous atrophy (NCVA), with various mechanisms of intestinal damage that include direct drug toxicity to the mucosa, immune effects, and disruption of intestinal homeostasis [6,7]. Drugs of most significant importance in the pathogenesis of NCVA include non-steroidal anti-inflammatory drugs (NSAIDs), angiotensin receptor blockers (ARBs) such as olmesartan, and immunosuppressants, including mycophenolate mofetil (MMF). NSAIDs specifically contribute to mucosal injury through systemic inhibition of prostaglandin synthesis, undermining the protective mechanisms of the gastrointestinal lining. This inhibition promotes chronic inflammation and mucosal damage, which may eventually lead to villous atrophy, particularly in long-term users or individuals with intrinsic vulnerabilities [20]. Given the prevalence of NSAID use, this underscores the importance of identifying individuals at increased risk for NCVA and implementing mitigation strategies, such as concomitant gastroprotective agents.

Olmesartan, a notable example in the ARB group, is associated with an enteropathy that mimics celiac disease. Such enteropathy is characterized by T-cell activation and inflammation, causing villous atrophy and lymphocytic infiltration of the intestinal mucosa. Clinical manifestations often include severe diarrhea, weight loss, dehydration, and even organ failure, with symptoms most prominent in Caucasians over 50 years of age [38,39]. The development of enteropathy secondary to olmesartan therapy usually occurs only after prolonged use of the drug, and withdrawal brings marked improvement, although recovery periods vary. Histological examination of olmesartan-induced enteropathy shows shortened intestinal villi and increased numbers of intraepithelial lymphocytes. Such a picture overlaps significantly with that of celiac disease, which can lead to diagnostic confusion [34]. To this end, a thorough review of the patient’s medication history is necessary to avoid misdiagnosis. In addition, it is important to investigate why some people, especially the elderly, are more susceptible to the disease. Factors such as diminished epithelial repair capacity or age-related immune changes may exacerbate the effects of ARB-induced villous damage but remain insufficiently investigated in published studies to date.

Mycophenolate mofetil and its enteric-coated variant, mycophenolic acid, represent another group of drugs strongly implicated in NCVA. These drugs, commonly prescribed to transplant recipients, impair the intestinal barrier function by promoting chronic inflammation and altering electrolyte transport. Such disruptions contribute to malabsorption and villous atrophy, significantly increasing the risk of graft loss and mortality in vulnerable patient populations [38,40]. The systemic impact of MMF-associated enteropathy is further exemplified by its influence on glucose absorption and chloride secretion, which underscores the extensive metabolic ramifications of such treatment [40]. The need for patient-specific dosing adjustments or alternative immunosuppressive therapies is critical to mitigate these adverse effects without compromising transplant outcomes. However, little research has been conducted into balancing immunosuppression and gastrointestinal safety to optimize treatment for transplant patients.

The pathophysiological mechanisms connecting these medications to NCVA frequently overlap, leading to diagnostic challenges that complicate patient care. For instance, olmesartan-associated villous atrophy often resembles celiac disease both histologically and symptomatically. Consequently, recognizing drug-induced causes requires systematic diagnostic approaches integrating histological findings, patient histories, and clinical presentations [25,38]. For example, distinguishing drug-induced enteropathies from autoimmune conditions may be facilitated by identifying unique histological markers like crypt hyperplasia or mucosal thickening, paired with the observation of symptomatic resolution after drug discontinuation. Current diagnostic frameworks, however, fall short in standardizing these evaluations, highlighting an area for future improvement. Advances in high-sensitivity molecular diagnostics can provide a valuable component in the differential diagnosis process and should be prioritized in future clinical trials.

Olmesartan cessation has been shown to improve clinical symptoms and histological findings, often within weeks to months [39]. However, severe cases may require adjunctive therapies, such as glucocorticoids or prolonged parenteral nutrition, to restore epithelial integrity and manage residual inflammation [38]. Similarly, MMF cessation leads to symptom relief in many cases, albeit at different times, depending on the extent of villi damage and the severity of underlying immune requirements [36]. Patients with long-term mucosal damage may require comprehensive nutritional rehabilitation, and systematic follow-up care is essential to ensure complete intestinal recovery.

The relationship between NCVA and the medications described above underscores the importance of awareness and education of clinicians about drug-induced enteropathy to facilitate its inclusion in the differential diagnosis. Patient histories remain essential in identifying suspicious cases, especially when symptoms mimic celiac disease or other forms of NCVA. Educating patients on the risks associated with these medications can minimize long-term complications and improve adherence to safer treatment regimens. Moreover, research into safer drug formulations with reduced gastrointestinal toxicity, particularly within immunosuppressive and antihypertensive drug classes, holds substantial potential for reducing the prevalence of drug-induced NCVA [40].

The current understanding of drug-induced enteropathy is limited by an incomplete exploration of its underlying mechanisms. While it is well-recognized that NSAIDs and ARBs influence inflammatory pathways, additional research into their specific molecular actions within the gut is essential. For example, a thorough understanding of the cytokine profiles associated with ARB-induced enteropathy may reveal particular biomarkers for early detection and provide further insight into therapeutic targets [17]. Similarly, patient-specific genetic and epigenetic factors may predispose specific individuals to develop NCVA when exposed to NSAIDs, MMF, or ARBs. Identifying such factors could enhance personalized medicine approaches, guiding safer prescription practices and preemptive interventions for at-risk patients.

In summary, drug-induced enteropathy plays a significant role in the pathogenesis of NCVA, with strong evidence pointing to NSAIDs, ARBs (including olmesartan), and immunosuppressants (such as MMF). Despite its rarity, olmesartan-induced enteropathy serves as a reminder of the importance of reviewing patients’ treatment histories to avoid misdiagnosis and unnecessary treatment. Future research into the molecular pathways and patient-specific susceptibilities underlying these conditions is necessary to improve diagnostic accuracy and therapeutic strategies.

### 4.2. Demographic and Environmental Factors

The literature shows that demographic factors have a significant impact on the etiology, course and prognosis of NCVA. At the same time, it has been observed that the influence of demographic factors, such as age, gender and ethnicity, on the incidence and course of NCVA varies according to the cause of disease development.

Age is one of the key factors associated with the etiology of NCVA, showing marked differences between the pediatric and adult populations [10]. In children, the most common causes of NCVA are inflammatory and infectious factors and immune-mediated disorders, while among adults, seronegative celiac disease forms and iatrogenic etiologies predominate [5,10]. In the pediatric population, early signs of NCVA associated with autoimmune enteropathy are observed as early as the first months of life. The literature shows that about 65% of cases of autoimmune enteropathy begin before 3 months of age [14]. In contrast, the typical manifestation of the disease is most often noted before the age of 6 months. In the adult patient population, the median age of diagnosis of autoimmune enteropathy is about 55 years, which may indicate a tendency for late detection of this pathology [1].

The results of currently available studies indicate that patient age at diagnosis is also a key and independent prognostic factor affecting the prognosis of patients with NCVA [1]. A multicenter study representing a 30-year follow-up of 261 patients with non-celiac enteropathies showed that advanced age at diagnosis was associated with significantly higher mortality (*p* < 0.001) [1]. Patients with NCVA diagnosed after age 60 were at particularly high risk. In the cited study, the mean age at which NCVA was diagnosed in the total study population (261 patients) was 49 ± 18 years, with significant differences in the time of diagnosis depending on the pathogenetic factor. The mean age of diagnosis of autoimmune enteropathy was 40 ± 20 years. The mean age of CVID diagnosis was 39 ± 15 years. It was also reported that CVID was diagnosed mainly in children between 1 and 5 years old and in young adults between 18 and 25 years old [13]. Drug-induced enteropathy, on the other hand, mainly affected the oldest patients, with a mean age of diagnosis of 66 ± 12 years. Idiopathic enteropathy was diagnosed most often in patients in the age range of 40–59 years (mean age 49 ± 17 years) [1]. Collagenous sprue was diagnosed primarily in elderly patients. In contrast, eosinophilic gastroenteritis was most common in men between the ages of 30 and 50 [13]. Schiepatti et al. also observed that older age at diagnosis similarly co-occurred with anemia, and clinical nonresponse and histologic nonresponse were significant independent predictors of mortality in the non-celiac villous atrophy patient population [1].

The influence of gender on the incidence and clinic of NCVA is less clear than for celiac disease, but some characteristic patterns are observed depending on the type of enteropathy and the age of onset of symptoms. Autoimmune enteropathy shows clear differences in gender distribution, depending on the age of onset. In the pediatric population, boys predominate, accounting for as many as 75% of cases [14]. This observation contrasts with data on adult patients, among whom (according to a European study) 62% of autoimmune enteropathy cases were female, with a median age of 52 years [12]. In contrast, in a multicenter study involving 261 patients with NCVA, women made up 55% of the study population (N = 144), indicating a relatively even gender distribution [1].

There are few publications in the available literature describing the effect of ethnicity on the occurrence or course of NCVA. One showed that eosinophilic gastroenteritis, which can secondarily lead to atrophy of the intestinal villi, most often affects Caucasians and to a much lesser extent Asians [13]. Of particular note are also observations of populations of Asian descent in which Enteropathy-Associated T-cell Lymphoma (EATL) is relatively more common [16]. Between 2000 and 2010, the incidence of EATL in Asian and Pacific Islander populations was 0.236 per million, compared to 0.101 in Caucasians, 0.107 in Blacks, and 0.128 in Native Americans and Alaska Natives. The relative risk for people of Asian descent compared with non-Asians was 2.32 (95% CI [1.39–3.69]; *p* = 0.002) [16]. In contrast, a U.S. study described significant ethnic differences in the prevalence of intestinal villous atrophy associated with celiac disease [15]. The cited authors, in an analysis involving 454,885 patients, observed the lowest prevalence of villous atrophy in people of South Indian (0%), East Asian (0.15%) and Hispanic (1.06%) descent compared to other Americans (1.83%) [15]. For those of Jewish and Middle Eastern descent, the prevalence of villous atrophy was significant compared to other Americans. Among the North Indian population, there was a trend toward a higher prevalence (2.04%); however, this was not a statistically significant difference [15].

Ethnic differences in the prevalence of NCVA have important practical implications. For patients of East Asian, South Indian and Hispanic descent who present with features of villous atrophy, etiologies other than classic celiac disease should be specifically considered [15]. At the same time, the higher prevalence of EATL in Asian populations suggests the existence of specific forms of enteropathy, unrelated to the classical mechanism of autoimmune celiac disease, but leading to serious cancer complications [16].

In summary, demographic factors have a significant impact on the etiology, course and prognosis of NCVA. Age also remains the most important prognostic factor. Older age at diagnosis correlates with poorer long-term prognosis. Gender differences in NCVA are less pronounced than in celiac disease, but characteristic patterns are observed depending on the type of enteropathy and age of onset of symptoms. Ethnicity, in turn, determines not only the prevalence of NCVA but also the prevalence of specific clinical phenotypes and disease etiologies. This indicates that selected populations require a specialized diagnostic approach due to the increased risk of atypical forms of enteropathy.

Other environmental factors described in the literature that play a significant role in both the development of NCVA and disease progression include chronic alcohol consumption. Case studies, such as the one described by Sjölund et al., have shown normalization of intestinal morphology and clinical improvement, including resolution of diarrhea and weight loss, after long-term alcohol abstinence. This reversibility suggests that alcohol-induced atrophy of intestinal villi may result from its direct toxicity to the mucosa, with secondary impairment of nutrient absorption. However, the specific biochemical pathways through which alcohol exerts these deleterious effects on the intestinal villi remain underexplored [41].

It is only known that the effects of alcohol on the intestinal barrier include changes in the expression of tight junction proteins, contributing to increased intestinal permeability and stimulation of inflammatory responses. This can significantly exacerbate mucosal damage and precipitate villous atrophy. These findings underscore the therapeutic potential of abstinence-based interventions in mitigating the effects of alcohol on intestinal health [41,42]. Nevertheless, further research into the dose-dependent effects of alcohol on molecular changes, particularly the modulation of zonulin and occludin expression, may provide a deeper understanding of the pathophysiological processes involved and help optimize therapeutic interventions for those affected by alcoholism. Yoseph et al. showed that in mouse models, septic mice intoxicated with alcohol had lower levels of occludin than septic mice intoxicated with water [41,43]. Moreover, examining the interplay between alcohol-induced permeability changes and gut microbial dysbiosis may offer insights into adjunctive therapies, such as probiotics, to counteract these effects.

One critical aspect of alcohol-induced villous atrophy is its reversibility, as evidenced in both clinical and histological improvements following cessation of alcohol use. This emphasizes the importance of identifying dietary and lifestyle contributors to NCVA and integrating behavioral interventions into management strategies for at-risk populations. However, the variability in individual responses to alcohol abstinence—likely influenced by genetic predispositions, nutritional status, and concurrent health conditions—highlights the need for personalized intervention plans. Such approaches could include tools to monitor the degree of intestinal recovery, like serial biopsies or biomarkers of intestinal barrier function, to modify the therapeutic plan effectively [41,42].

Chronic alcohol consumption may also exacerbate oxidative stress and impair mucosal immune responses, complicating the recovery of villous atrophy. Oxidative stress, characterized by excessive production of reactive oxygen species (ROS), destabilizes cellular components and propagates epithelial damage, disrupting the structural integrity of the villi. At the same time, suppression of mucosal immune function weakens intestinal defense mechanisms, increasing susceptibility to infection and further damage. Data available in the literature suggest that antioxidant therapy (e.g., glutathione supplementation) may positively affect alcohol-induced damage to the intestinal mucosa. The study of antioxidant therapies, such as glutathione supplementation, may represent a new avenue in alleviating alcohol-related intestinal damage; however, evidence for such interventions remains preliminary and requires further observational studies [41,44,45].

Bacterial toxins, arising from infectious agents or contaminated food sources, also contribute significantly to the development of villous atrophy by disrupting intestinal epithelial integrity. In conditions like tropical sprue, bacterial toxins trigger chronic inflammation and alter gut microbiota balance, resulting in persistent damage to the villi. The pathophysiological role of these toxins is further complicated by their ability to modulate immune responses, often inducing cytokine release that exacerbates mucosal injury. Addressing these factors requires targeted medical treatments and systemic public health strategies to improve water quality, food safety, and access to healthcare in affected regions [25,46].

Tropical sprue, a geographically specific condition observed in tropical climates, is a well-documented example of environmental factors contributing to NCVA. Its multifactorial etiology includes bacterial dysbiosis, nutrient malabsorption, and substandard sanitation, all of which exacerbate intestinal damage and lead to villous atrophy. Effective treatment has traditionally involved long-term antibiotic regimens, such as tetracycline, and folate supplementation to address coexistent micronutrient deficiencies. This combination has demonstrated significant efficacy in symptom resolution and villous regeneration [25,47,48]. Nevertheless, the persistence of cases in endemic regions highlights the need for enhanced public health interventions to address underlying environmental determinants, such as promoting hygiene and improving nutritional access.

The role of bacterial toxins in inducing villous atrophy underscores the complex interplay between host immune responses and environmental exposures. Chronic environmental conditions, such as inadequate sanitation, may perpetuate cycles of bacterial infections and sustained inflammation, necessitating more effective public health policies. Moreover, understanding the modulatory effects of gut microbiota on the host immune system could lead to innovative therapies to restore microbiome balance and reduce susceptibility to toxin-induced intestinal damage. Research into using probiotics and prebiotics as adjunctive interventions in such populations could provide promising avenues for future management strategies [25,49,50].

Tropical sprue exemplifies how lifestyle and environmental conditions can exacerbate NCVA, particularly in populations with limited access to clean water and healthcare resources. The synergistic contributions of chronic bacterial infections and malnutrition illustrate the critical role that environmental factors play in disease pathogenesis [17,25]. Addressing these issues requires global efforts to implement sustainable development practices, particularly sanitation and nutrition. These measures could significantly reduce the burden of tropical sprue and related conditions, facilitating disease prevention alongside medical treatment initiatives.

Resolution of tropical sprue symptoms with antibiotic therapy emphasizes the importance of targeting local environmental conditions, such as sanitation improvements and nutritional support, in conjunction with medical interventions. However, the persistence of endemic cases suggests gaps in current approaches to prevention and treatment. Additionally, the condition raises questions regarding genetic predispositions that may amplify individual susceptibility to environmental triggers. Such gene–environment interactions warrant deeper exploration to identify high-risk groups and develop precision-based public health strategies [25,48].

Environmental enhancements, such as access to clean water and improved food hygiene, remain essential to mitigating the prevalence of NCVA in regions prone to tropical sprue and similar conditions. These efforts highlight the broader public health implications of environmental factors in gastrointestinal diseases, underlining the need for interdisciplinary collaboration between healthcare providers, policymakers, and community organizations. Furthermore, emerging studies have suggested a potential role for epigenetic modifications, including microRNAs’ dysregulation, in mediating the effects of environmental exposures on intestinal health [51]. This could open new avenues for targeted interventions that address underlying molecular mechanisms.

As seen in conditions like tropical sprue, gene–environment interactions underscore the interplay between genetic predisposition and environmental triggers in NCVA development. This interplay is likely mediated by epigenetic factors that influence gene expression profiles in response to external stimuli. Investigating these interactions could elucidate predictive biomarkers for NCVA susceptibility, enabling earlier identification and tailored interventions. Importantly, epigenetic profiles may also explain variability in clinical manifestations, underscoring the potential for personalized diagnostic and therapeutic strategies based on individual genetic and environmental contexts.

Environmental stressors, such as infections and dietary toxins, influence gut microbiota composition and related epigenetic pathways, contributing to the development of villous atrophy. For example, food additives like emulsifiers and gluten substitutes may damage the intestinal epithelium by disrupting tight junction proteins and promoting inflammation, particularly in genetically susceptible individuals [20,38]. Further research into the long-term impacts of dietary components on gut health could improve safety guidelines and inform preventative dietary recommendations for vulnerable populations.

While some studies suggest that food additives exacerbate NCVA in predisposed individuals, there is a need for stricter regulatory oversight and improved labeling practices to minimize inadvertent exposure to potentially harmful substances. Identifying specific molecular pathways by which these additives disrupt barrier function could inform dietary recommendations and therapeutic interventions. Nutritional counseling and personalized dietary plans must be prioritized for individuals with NCVA, as they may play a critical role in mitigating disease progression and severity [17,52].

There is a growing body of evidence in the scientific community highlighting the role of microplastics in the pathogenesis of various diseases. Environmental contamination with microplastics has increased as a result of rising plastic consumption and waste generation. It has been noted that microplastics can enter the human body through contaminated food and water, as well as via inhalation of airborne particles [53]. The EFSA Panel on Contaminants in the Food Chain reports that microplastic particles smaller than 150 μm can be transported across the intestinal epithelium, and particles smaller than 5 μm can penetrate intestinal cells and accumulate in internal organs [53,54]. In murine models, exposure to microplastics has been shown to compromise intercellular integrity of the intestinal wall and damage the mucosal barrier [53,55].

Other animal studies have also indicated that exposure of the gut microbiota to microplastic particles may lead to intestinal dysbiosis [53]. Preclinical evidence suggests that microplastics promote immune system activation and are associated with intestinal inflammation. Histological analyses of tissues collected from animal models have demonstrated that microplastic-induced inflammation results in edema, crypt hyperplasia, and disruption of microvilli structures throughout the gastrointestinal tract, from the duodenum to the colon [56,57].

The results of studies available in the scientific literature also indicate a significant impact of environmental pollutants on the development and course of enteropathies [58,59]. Although direct studies linking specific pollutants to NCVA are still limited, the mechanisms by which environmental pollutants may contribute to the development and worsening of intestinal diseases, including NCVA, are recognized. Studies reveal complex mechanisms by which different classes of environmental pollutants affect the gastrointestinal tract. Air pollutants can affect the gastrointestinal tract via the following ways:

- Inducing systemic inflammation;
- Increasing intestinal permeability;
- Disrupting the balance of intestinal microflora;
- Inducing oxidative stress and epithelial cell damage.

According to a study by Kou et al., long-term exposure to air pollutants has a significant impact on increasing the risk of gastrointestinal (GI) diseases [58]. The cited authors showed that long-term exposure to particulate matter (PM2.5 and PM10), sulfur dioxide (SO_2_), nitrogen dioxide (NO_2_) and carbon monoxide (CO) was significantly correlated with a higher risk of GI diseases. Particulate matter with an aerodynamic diameter ≤ 2.5 µm (PM2.5) was associated with a 38% increase in the risk of gastrointestinal diseases (HR = 1.38; 95% CI [1.33–1.44]). Its mechanism of action included induction of oxidative stress, activation of inflammatory responses and damage to the intestinal barrier. Similar relationships were found for PM10 (≤10 µm), which increased risk by 31% (HR = 1.31; 95% CI [1.26–1.36]). Its impact was attributed to disruption of the intestinal microbiota and increased systemic inflammation, which could lead to weakened intestinal mucosal integrity. The strongest association with gastrointestinal disease risk was noted for sulfur dioxide (SO_2_), for which the risk ratio was HR = 1.74 (95% CI [1.68–1.81]). This pollutant had a negative effect on the intestinal mucosa by increasing its permeability and inducing inflammatory reactions. Nitrogen dioxide (NO_2_) also significantly increased the risk of gastrointestinal diseases (HR = 1.21; 95% CI [1.17–1.25]). The mechanism of action involved permeation into the general circulation after inhalation and generation of chronic inflammation and oxidative stress within the gastrointestinal tract. Carbon monoxide (CO) increased the risk of GI disorders by 48% (HR = 1.48; 95% CI [1.42–1.54]). Chronic exposure to this compound led to mitochondrial dysfunction, impaired cellular respiration and the development of inflammation, as well as adverse changes in the composition of the intestinal microflora.

The effect of environmental pollutants on the intestinal microbiota through the induction of dysbiosis needs special mention [59]. Disruption of microbial homeostasis induced by chronic exposure to environmental pollutants results in abnormalities in energy metabolism, impaired nutrient absorption, impaired immunity, and increased levels of pro-inflammatory cytokines and oxidative stress [59]. The intestinal microbiota also plays a key role in the metabolism and detoxification of xenobiotics, chemicals that are foreign to the body. Its disruption can result in prolonged exposure to toxic chemicals and increased susceptibility to their harmful effects. Importantly, dysbiosis of the gut microbiota is also recognized as an important mediator in the relationship between exposure to environmental pollutants and the increasing incidence of early-onset colorectal cancer [59].

Environmental pollutants, including heavy metals and pesticides, represent an additional concern due to their capacity to exacerbate NCVA through gut microbiota disruption and persistent low-grade inflammation. Experimental evidence suggests that pollutants such as arsenic and lead impair epithelial integrity and alter immune responses, contributing to villous atrophy. Understanding these mechanisms is critical for guiding environmental health policies to reduce pollution-related risks for gastrointestinal diseases [60,61].

In animal models, elevated cadmium concentrations have been shown to lead to increased accumulation of this heavy metal in the body, resulting in histopathological alterations within the intestine. These changes included muscular layer damage, villous atrophy, excessive mucus production, and an increased number of goblet cells. Impaired intestinal immunity was also observed in fish exposed to the toxic effects of cadmium [62].

It has been demonstrated that cadmium, even at low concentrations, may negatively affect the human gut microbiome, induce oxidative stress, and trigger an inflammatory response within the intestine [63].

Glyphosate is a widely used chemical in agricultural industries. It functions as a herbicide, enabling the control of weeds in crop fields [64,65]. In a study conducted by Shaimaa et al. on rats exposed to glyphosate, histopathological changes were observed in the intestinal mucosa of the exposed group compared to the control group. These changes included villous deformities, villous atrophy, and alterations in the lamina propria, such as its detachment from the basement membrane. These abnormalities may suggest similar pathological changes in humans exposed to glyphosate [65].

Geographic differences in the prevalence and severity of the disease indicate the importance of environmental toxins in the pathogenesis of NCVA. Some populations have been observed to have a disproportionately higher risk due to regional exposure. Tackling pollution-induced villus atrophy requires a multidisciplinary approach combining environmental science, gastroenterology, and public health expertise. Preventive measures such as stricter environmental regulations and community education campaigns may reduce the prevalence of NCVA in highly exposed populations and improve health outcomes.

Emerging evidence suggests the involvement of epigenetic mechanisms, including microRNA dysregulation, in the impact of environmental factors on intestinal health. For example, microRNA modifications associated with intestinal permeability and immune response may underlie host susceptibility to NCVA. Investigating these molecular pathways could lead to novel diagnostic markers and therapeutic targets tailored to the unique challenges posed by environmentally driven villous atrophy [51].

In conclusion, environmental factors are important in the etiology and progression of NCVA. Environmental factors can significantly influence disease clinics and therapeutic outcomes, particularly when co-mingling with genetic and epigenetic factors. A comprehensive approach integrating clinical management with public health interventions is needed to address the multifaceted challenges associated with these factors.

### 4.3. Metabolic Disorders

Metabolic disorders represent a unique and multifaceted group of pathogenetic factors in NCVA, often rooted in structural and functional abnormalities of the intestinal epithelium and deregulated nutrient absorption. Congenital enteropathy exemplifies this, characterized by intrinsic epithelial defects such as hypoplastic villus atrophy, which severely disrupt nutrient bioavailability pathways. Epithelial changes, including the absence of a brush border and increased numbers of lysosome-like inclusions, correlate with decreased glucose and sodium absorption, leading to symptoms such as severe malnutrition and dehydration [17,66]. These symptoms persist despite dietary support, underscoring the critical role of epithelial integrity in maintaining intestinal absorption. The specific mechanisms underlying epithelial disruption remain inadequately explored, suggesting an urgent need for research into molecular pathways to enhance diagnostic precision and therapeutic interventions.

Persistent villous atrophy in congenital enteropathy highlights the failure of the intestine to adapt functionally despite external support. The absence of inflammation, typically associated with immune-mediated conditions, contrasts sharply with the intrinsic epithelial defects observed in such cases. Structural anomalies like crypt hypoplasia and the lack of increased inflammatory cells suggest that the pathogenesis is non-immune. This understanding necessitates a paradigm shift toward therapeutic strategies targeting the epithelial abnormalities rather than immune system modulation [66]. For example, molecular therapies to restore epithelial function may hold promise for treating metabolic NCVA. Despite the significant advances in understanding congenital enteropathy, the limited availability of targeted therapies highlights the necessity for more comprehensive research into epithelial repair mechanisms.

Total parenteral nutrition (TPN) is often indispensable for individuals with congenital enteropathy due to their inability to absorb nutrients effectively. However, even with aggressive nutritional support, complications such as progressive failure to thrive and systemic consequences often lead to severe outcomes, including high mortality rates [17,66]. These cases illustrate the limitations of current interventions and underline the critical need to develop strategies addressing the root causes of malabsorption rather than merely compensating for its effects. Developing new therapeutic approaches, such as epithelial stem cell therapy, could revolutionize treatment in these high-risk populations, but such approaches remain experimental.

Secondary to structural and metabolic impairments, chronic nutrient deficiencies such as vitamin B12 frequently worsen the clinical picture in NCVA. This is often seen in conditions involving ileal resection or bacterial overgrowth, both of which interfere with B12 absorption. The impairment of DNA synthesis and cellular repair mechanisms due to B12 deficiency exacerbates villous damage and can lead to systemic complications such as anemia and neuropathy [17,67]. Addressing these secondary deficiencies through early supplementation and consistent monitoring of nutritional status can mitigate systemic effects. Future studies could focus on optimizing absorption methods, particularly in patients with severe intestinal resection.

The dual threat posed by ileal resection is evident, as it disrupts vitamin B12 reabsorption and impairs bile acid recycling. Accumulated bile acids may irritate the intestinal mucosa, inducing inflammation and perpetuating villous damage and causing debilitating diarrhea. The chronic irritation further contributes to metabolic dysfunction, compounding malabsorption and systemic effects. Early therapeutic interventions, such as bile acid sequestration therapy, have shown potential in managing these symptoms and preventing further damage. However, variability in patient responses to these therapies calls for a more personalized approach, integrating factors such as genetics and the degree of intestinal resection [17,68,69].

Bacterial overgrowth and dysbiosis (altered microbiota) significantly exacerbate metabolic challenges in NCVA by further impairing nutrient digestibility and bioavailability. The disruption of microbial homeostasis amplifies villous atrophy, underscoring the interconnectedness of metabolic and microbial pathways. Antibiotics and probiotics have provided relief, improving microbial balance and nutrient absorption [17]. However, the long-term efficacy and safety of such treatments remain poorly understood. Research into the broader impact of the microbiome on gut repair mechanisms may pave the way for more precise therapies aimed at restoring eubiosis.

Tropical sprue is an example of the interplay between metabolic dysfunction and environmental influences, with chronic bacterial infections and malnutrition driving villous atrophy. The condition is marked by the combined effects of bacterial toxins, persistent inflammation, and nutrient deficiencies, particularly folate and vitamin B12, impairing metabolic balance [17,25]. The success of folate supplementation alongside antibiotics in reversing villous damage demonstrates the critical role of simultaneously addressing metabolic and structural deficits. However, incomplete responses in some patients highlight the need for more precise treatment strategies, potentially incorporating individualized metabolic profiling [17,25,70,71].

Bacterial toxins in tropical sprue appear to inhibit basic metabolic pathways such as folate synthesis and uptake, further exacerbating epithelial damage and hindering recovery. Targeted use of folate supplements has shown promise in alleviating these metabolic abnormalities, but the variability in outcomes among individual patients suggests a deeper, possibly genetic, factor influencing treatment efficacy. This observation points to the necessity for research into genetic and epigenetic variations underlying individual susceptibility to bacterial toxins, which could inform more personalized therapeutic interventions [17,25,47,72].

The response to antibiotic therapy in tropical sprue is inconsistent, with some individuals showing rapid improvement while others experience lingering symptoms. This variability may stem from differences in underlying metabolic pathways affected by the disease [17,48]. Further research into these differences could refine treatment strategies, ensuring that therapies are optimized for each patient’s metabolic profile.

Immunosuppressive therapies commonly prescribed in transplantation significantly contribute to drug-induced metabolic challenges that exacerbate NCVA. Mycophenolate mofetil, for example, disrupts intestinal barrier function through impaired glucose absorption and altered electrolyte transport, aggravating malabsorption and villous atrophy. The resulting metabolic dysfunction increases the risk of nutrient deficiencies and systemic complications, necessitating carefully balanced immunosuppressive regimens. However, limited research into patient-specific dosing and alternative immunosuppressants underscores the need for tailored therapeutic approaches that protect intestinal health without compromising transplant outcomes [17,38,40,73].

Immunosuppressive therapies also highlight vulnerabilities related to underlying genetic predispositions, particularly in transplant populations. The challenge of balancing immunosuppression with maintaining intestinal homeostasis illustrates the need for personalized regimens to mitigate villous damage while preserving graft function. Emerging research into genetic markers of drug sensitivity could guide safer prescription practices, improving gastrointestinal and systemic outcomes [17,74].

Nutritional interventions, including TPN and micronutrient supplementation, remain key in treating drug-induced NCVA. These therapies mitigate long-term metabolic consequences and provide critical support to patients experiencing severe malabsorption. However, relying solely on these interventions will not address the root causes of villous atrophy, pointing to the need for a broader focus on preserving intestinal integrity and preventing further metabolic deterioration [17,75,76].

The interaction between genetic predispositions and environmental triggers significantly influences the metabolic pathways underlying NCVA. Emerging research on dysregulated microRNAs and epigenetic modifications offers insights into how these factors may disrupt cellular repair processes, exacerbating villous atrophy [17,20]. Identifying molecular pathways involved in these gene–environment interactions could open new diagnostic and therapeutic avenues, tailoring strategies to individual patient profiles.

Experimental studies on microRNA modulation have identified potential therapeutic targets for restoring metabolic balance and epithelial health in NCVA. These findings illustrate the growing potential of epigenetic therapies in addressing not just metabolic outcomes but also the broader pathophysiological processes driving villous atrophy [17]. However, these therapies remain early, requiring more comprehensive investigation to transition into clinical applications.

The complexity of NCVA’s metabolic underpinnings emphasizes integrating genetic, environmental, and metabolic insights into diagnostic and therapeutic strategies. A multidisciplinary approach will be critical for addressing the multifaceted challenges posed by these metabolic disorders to advance patient care and improve outcomes [17].

## 5. Diagnostic Approaches

**Figure 2:**
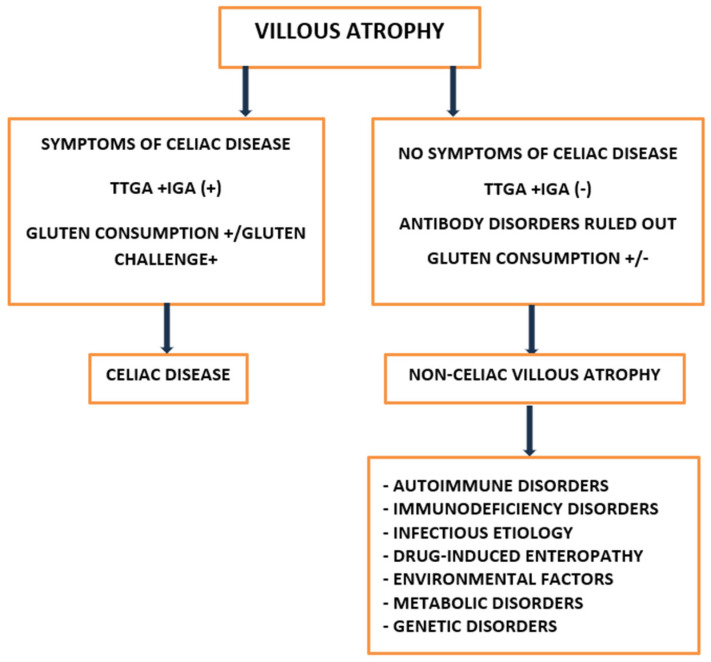
A brief outline of the differential diagnosis of celiac and non-celiac villous atrophy; IgA: immunoglobulin A, TTGA: tissue transglutaminase antibodies.

The multifaceted nature of non-celiac villous atrophy necessitates a thorough diagnostic approach to distinguish between its diverse causes. This section delves into the critical methodologies employed to identify and differentiate conditions leading to villous atrophy, encompassing histopathological assessments, serological testing, and essential differential diagnosis strategies. By integrating these diagnostic tools, clinicians can accurately determine the underlying etiology, informing targeted treatment strategies and enhancing patient management within the broader context of gastrointestinal health. A brief outline of the differential diagnosis of celiac and non-celiac villous atrophy is presented in Figure 2.

### 5.1. Histopathological Assessment

Histopathological assessment remains the cornerstone of diagnosing villous atrophy, offering vital insights into the underlying etiologies by identifying specific patterns of intestinal damage. The morphological analysis focuses on varying degrees of villous atrophy, crypt hyperplasia, and intraepithelial lymphocytosis, which, when evaluated collectively, help correlate histological findings with potential clinical causes. Degrees of villous atrophy, categorized as partial, subtotal, or total, are critical for refining diagnostic pathways and establishing probable conditions responsible for intestinal damage [17]. Crypt hyperplasia, a characteristic elongation of the crypts accompanied by increased mitotic activity, often reflects compensatory mucosal repair mechanisms and highly indicates active mucosal damage [24,77,78]. Furthermore, intraepithelial lymphocytosis, defined by an elevated lymphocyte count of over 25 intraepithelial lymphocytes per 100 enterocytes, serves as an essential histological marker, particularly in distinguishing celiac disease. However, its presence alone is not pathognomonic and requires careful contextual interpretation alongside other histological and clinical features [17,79]. These observed changes underscore the significance of integrating histological findings with clinical data to effectively delineate the causes of villous atrophy with overlapping features.

Within the context of celiac disease, the Oberhuber classification (A1—non-atrophic; B1—atrophic, villous–crypt ratio < 3:1; B2—atrophic, no villi can be detected) refines the original Marsh grading system (IIIa—partial villous atrophy; IIIb—subtotal villous atrophy; IIIc—total villous atrophy) by delineating subtypes of villous atrophy to assess the severity of mucosal degradation. Such classifications detail the progressive disruption of villous architecture and guide the identification of cases that span partial to total atrophy [80,81,82]. Nonetheless, it is essential to recognize that this classification is not specific to celiac disease, as histological patterns resembling Marsh 3b and 3c lesions are observed in other conditions, such as autoimmune enteropathy and drug-induced enteropathy [24,81].

Autoimmune enteropathy, for instance, is distinguished histologically by crypt apoptosis and goblet cell depletion, features that diverge notably from gluten-mediated damage patterns [17]. Similarly, olmesartan-induced enteropathy mimics celiac disease histologically but is distinguishable through the absence of serological markers associated with celiac disease and a suggestive medication history [25,39]. The diagnostic challenge these overlaps present underscores the necessity of a multidimensional approach that incorporates histopathological data, clinical evaluations, and serological testing to avoid misdiagnosis.

Infectious etiologies such as giardiasis or Whipple’s disease further exemplify the histological complexities associated with villous atrophy. The presence of crypt hypertrophy combined with villous atrophy in these conditions highlights the diversity of histological manifestations that are not exclusive to celiac disease [24]. Supplementary investigations, such as stool antigen assays or endoscopic identification of parasites, are thus essential for confirming infectious causes [25]. Chronic inflammatory conditions like Crohn’s disease, which typically exhibit transmural inflammation and granuloma formation, offer another distinct histopathological profile that diverges from the mucosal-level changes predominantly seen in celiac disease [17]. Such contrasts in histopathological features underscore the role of pathogenesis in uncovering disease-specific patterns that guide further diagnostic measures and treatment strategies.

Adequate biopsy sampling techniques are critical for accurate histological diagnosis of villous atrophy. Obtaining at least four duodenal specimens is widely recommended to reduce the likelihood of sampling error, particularly in conditions like early celiac disease and focal drug-induced enteropathies, where patchy involvement may obscure findings [17]. Biopsies from multiple sites, including the duodenal bulb and distal duodenum, increase diagnostic sensitivity and provide a more comprehensive representation of intestinal damage. Proper biopsy orientation and preparation are also essential, as interpretative errors arising from misaligned villi or other technical artifacts can lead to false assessments of atrophy [25]. Collaborative efforts between gastroenterologists and pathologists further enhance the precision of diagnoses by correlating histological observations with the patient’s clinical presentation, dietary history, and serological outcomes [24]. Pathologists play a critical role in identifying specific inflammation patterns or histological anomalies, such as crypt apoptosis in autoimmune conditions, guiding subsequent diagnostic investigations, and uncovering underlying etiologies for cases of non-celiac villous atrophy [19].

Non-celiac villous atrophy demonstrates various histological patterns based on its etiology, requiring nuanced interpretation to distinguish it from gluten-mediated damage. Autoimmune enteropathy, for instance, is marked by distinct crypt destructiveness and apoptosis, which differ significantly from the crypt hyperplasia typical of celiac disease [17,83]. Similarly, olmesartan-associated enteropathy presents with villous shortening and lymphocytic infiltration. Still, it lacks the immune response to gliadin seen in celiac disease, necessitating a careful review of patient medication history for diagnosis [25,39]. Infectious causes of villous atrophy, such as giardiasis, may show crypt hypertrophy alongside atrophic changes and require confirmatory diagnostic tools, such as stool analysis or direct visualization of the pathogens, for definitive diagnosis [24]. Chronic inflammatory conditions, including Crohn’s disease, produce unique histological markers like granulomas that aid in differentiating these cases from celiac disease, even when overlapping symptoms such as diarrhea are present [17,84]. These distinctions highlight the importance of leveraging histological findings within the broader clinical and diagnostic data context to ensure accurate identification and treatment of villous atrophy.

The role of histopathology also extends to reevaluating diagnostic criteria for atypical presentations of celiac disease. A study by Kurppa et al. demonstrated that children who tested positive for endomysial antibodies (EmA) but exhibited normal villous morphology could still benefit from a gluten-free diet [85]. These children, who lacked villous atrophy, showed significant gastrointestinal symptom improvement and antibody normalization after dietary changes, highlighting the potential for serology-guided interventions even in cases without overt histological damage. This finding challenges traditional diagnostic paradigms that rely heavily on the presence of villous atrophy for celiac disease confirmation, suggesting that early treatment based on serological data alone could prevent disease progression [85]. The research underscores the importance of incorporating serological findings into histopathological assessments, particularly in pediatric populations, where disease manifestations may differ from those in adults. It also raises crucial questions about whether similar diagnostic flexibility could apply to adult populations with histologically non-celiac conditions resembling celiac disease but lacking serological markers.

Recent advancements in molecular pathology have bolstered the histopathological assessment of villous atrophy by integrating histological findings with genetic and molecular markers. For example, monogenic disorders like CTLA4 haploinsufficiency, which contribute to autoimmune enteropathy, are increasingly identified through genetic testing, allowing precise diagnoses in refractory or atypical cases [17,86]. Similarly, mutations in pathways such as nuclear factor kappa B or IL-10 have clarified mechanisms of immune dysregulation underlying specific forms of inflammatory bowel diseases and non-celiac villous atrophy. The inclusion of next-generation sequencing technologies enables the stratification of patients into specific genetic subtypes of enteropathies, such as IL-10 receptor mutations in inflammatory conditions, thereby guiding targeted therapeutic approaches [17,87]. Broadening the application of molecular diagnostics could significantly enhance the precision of identifying and managing complex or overlapping causes of villous atrophy.

A significant limitation of current histopathological methods is the reliance on subjective interpretations, which can lead to inter-observer variability and inconsistent diagnoses. Standardized frameworks like the Corazza and Villanacci classification aim to address these issues by offering a simplified approach that categorizes mucosal changes into non-atrophic, partially atrophic, and atrophic patterns [80,82]. These classifications ensure greater consistency in biopsy interpretation across diverse clinical settings and align diagnostic criteria more closely with clinical decision-making. Future advancements in digital pathology, such as AI-driven biopsy analysis, hold the potential to further mitigate observer bias by offering consistent and objectively derived histological assessments [17]. However, efforts to validate and integrate simplified frameworks and emerging technologies into clinical guidelines remain limited, highlighting an area for improvement in the diagnostic process [24].

Despite its challenges, histopathological assessment remains an indispensable tool for diagnosing villous atrophy and differentiating its diverse etiologies. Advances in molecular diagnostics and the development of standardized classifications promise to enhance diagnostic precision, while collaborative, multidisciplinary efforts will continue to play a vital role in achieving accurate and effective outcomes.

### 5.2. Serological Testing

Serological testing plays a pivotal role in the diagnostic process of villous atrophy, particularly for gluten-mediated conditions like CD. Tissue transglutaminase IgA (tTG-IgA) and anti-endomysial antibodies (EMA) are highly sensitive and specific markers for CD and remain foundational diagnostic tools. These markers are particularly valuable in patients with classic CD symptoms, such as chronic diarrhea and malabsorption. However, the absence of these markers is a defining feature in seronegative enteropathies (SNEs), emphasizing the importance of their presence or absence as a primary step in the differential diagnosis of villous atrophy cases [17,88]. Despite their high reliability, discrepancies in interpretation or test performance can arise, necessitating standardization in laboratory testing and greater attention to pre-analytical variables.

The capacity of serological testing to differentiate CD from other causes of villous atrophy underscores its utility, but it also highlights its limitations. While tTG-IgA and EMA are instrumental for identifying gluten-driven damage, their absence in cases like drug-induced enteropathy or autoimmune enteropathy requires careful integration with histological and clinical findings to avoid misdiagnosis. This reliance on supplementary diagnostic methods indicates the need for a comprehensive diagnostic framework combining serological data with histopathological and patient history analyses [17,89,90].

Serological testing is a cost-effective and non-invasive approach, but its utility is constrained by its specificity to gluten-mediated immune responses. This limitation leaves non-CD causes of villous atrophy undetectable, necessitating alternative diagnostic methods when serology results are negative. Relying too heavily on serology without follow-up investigations can lead to diagnostic pitfalls, particularly in complex cases of villous atrophy with overlapping clinical features [17]. Advancements in imaging and biomarker technologies could fill this gap, allowing for a more robust diagnostic evaluation in cases where serology fails to provide conclusive evidence of the underlying cause.

Variability in the sensitivity and specificity of serological testing across different laboratory settings raises concerns about standardization. Ensuring consistent calibration of testing procedures is essential to maintain diagnostic reliability. Emerging technologies, such as next-generation sequencing-based assays, promise enhanced diagnostic accuracy by detecting novel or atypical markers. These advancements could revolutionize serological testing, particularly in identifying undiagnosed conditions contributing to villous atrophy [17,81].

IgA deficiency is an important confounding factor in tTG-IgA and EMA testing, as its presence makes these markers unreliable. Alternative markers, such as deamidated IgG gliadin peptide (DGP-IgG), offer a practical solution, especially for screening individuals with diagnosed IgA deficiency. However, these alternatives are generally less sensitive and should be used with a broader diagnostic evaluation. Considering the high prevalence of IgA deficiency among CD patients, routine screening for IgA levels before initiating serological tests is critical to avoid false-negative results and streamline the diagnostic process [25]. This necessity highlights the importance of clinical awareness and screening standardization to improve diagnostic pathways.

The detection and reliability of DGP-IgG tests may vary across populations, given the genetic differences in CD prevalence and IgA deficiency. Regional and population-specific studies could provide valuable insights into the test’s effectiveness, particularly in underserved or low-resource settings where diagnostic accuracy is crucial. Tailored diagnostic protocols may minimize diagnostic disparities, ensuring equitable healthcare delivery. Integrating a multidisciplinary approach is key to accurately diagnosing atypical cases of villous atrophy, such as those resulting from serology-negative conditions. Collaboration with immunologists could help validate suspected IgA deficiency, ensuring comprehensive evaluations in patients with negative serological results. This approach minimizes inaccurate diagnoses and provides a more straightforward pathway for identifying conditions like SNCD, which requires histological correlation and dietary intervention for confirmation [25,91,92].

Diagnosing SNCD among SNEs necessitates histopathological evidence and documented clinical response to a gluten-free diet. A confirmed diagnosis depends on significant histological improvement following dietary changes [17]. This process underscores the insufficiency of serology in addressing SNE cases, highlighting the diagnostic complexity of villous atrophy in clinical practice. Clinicians must recognize the diagnostic challenge SNCD presents to avoid delays, as delayed diagnoses often result in extended periods of malabsorption and nutritional deficiencies, worsening patient outcomes.

Approximately 9% of individuals with villous atrophy are diagnosed with SNCD, underscoring its clinical importance despite its relatively low prevalence. The absence of serological markers in these cases frequently leads to delayed recognition. Early suspicion and prompt initiation of dietary trials are critical for improving patient outcomes [3]. Such cases highlight the need for developing more sensitive diagnostic tests for SNCD, enabling earlier identification and treatment.

Crypt hyperplasia and villous atrophy, coupled with symptomatic relief following adherence to a gluten-free diet, remain pivotal indicators in diagnosing SNCD. Ongoing histological reassessment ensures accurate monitoring of dietary adherence and confirms therapeutic efficacy. This process also helps avoid misclassifications in cases mimicking other conditions, such as autoimmune or drug-induced enteropathy. Regular observation of the patient’s clinical condition and histologic reassessment provide a framework to ensure that these overlapping conditions are not misdiagnosed as SNCD.

Non-celiac causes of villous atrophy often lack definitive serological markers, posing a significant diagnostic challenge. For example, drug-induced enteropathies, including those caused by olmesartan, mimic celiac disease histologically but lack relevant serological findings. This necessitates thorough clinical evaluations, including a detailed review of patient medication history, to ensure accurate differentiation and diagnosis [5,39]. The absence of serological tools for such cases highlights the critical role of patient histories in establishing diagnoses and underscores the need for enhanced diagnostic pathways.

In autoimmune enteropathies, testing for anti-enterocyte antibodies offers some utility, albeit with limited sensitivity, as only approximately 50% of cases are positive for these markers. When specific antibody testing is inconclusive, genetic testing for mutations, such as CTLA4 haploinsufficiency, provides a necessary diagnostic clarification. This integration of molecular diagnostics with serology underscores the importance of a multifaceted approach in diagnosing autoimmune causes of villous atrophy [17].

Infectious etiologies of villous atrophy, such as Whipple’s disease or giardiasis, lack specific serological markers, necessitating alternative diagnostic methods like stool analysis or direct tissue staining. Such diagnostic adjuncts ensure accurate pathogen identification to guide treatment [25]. The absence of serology-based tools for infectious causes highlights the crucial role of microbiological investigations in the broader diagnostic framework.

Relying on patient histories to assess environmental exposures, infections, or medication use demonstrates the limitations of serology in diagnosing non-celiac villous atrophy. However, a structured diagnostic approach that begins with serological testing before incorporating histological, microbiological, and genetic evaluations minimizes diagnostic ambiguities and optimizes accuracy. This structured approach highlights the importance of maintaining a broad differential diagnosis when interpreting equivocal or negative serological findings [24].

Conditions such as CVID further illustrate the limitations of serologic testing, in which immune dysregulation interferes with antibody production, often leading to false-negative results. In such cases, alternative diagnostic methods, including immunoglobulin profiling and biopsy analysis, are essential to ensure accurate classification [25]. This demonstrates the need for a robust integration of immunology and gastroenterology in the diagnostic process.

Emerging technologies, such as next-generation immunological assays, could transform current diagnostic practices by detecting low-abundance or atypical antibodies in conditions like CVID. These advancements offer opportunities to enhance diagnostic reliability in challenging cases characterized by seronegativity [17]. However, translating these technologies into widespread clinical practice requires further development and evidence of efficacy.

Histological patterns and clinical symptoms remain primary diagnostic indicators in CVID-associated enteropathy cases, given the frequent failure of serological testing. The early introduction of immunoglobulin replacement therapy plays a vital role in managing these cases, often reducing gastrointestinal complications and resolving villous damage. This emphasizes the importance of prompt recognition and intervention to prevent disease progression [18].

Advancements in molecular diagnostics, including genetic testing for monogenic disorders like CTLA4 haploinsufficiency, complement traditional methods and serve as valuable tools in seronegative villous atrophy cases. The combination of molecular and serological analyses offers greater clarity in complex diagnostic scenarios, emphasizing the importance of a cohesive diagnostic framework [17,86,93]. This underscores the necessity of continually revising diagnostic guidelines to address emerging complexities in conditions resembling CD but lacking serological markers.

In conclusion, serological testing remains central to diagnosing celiac disease. Yet, its limitations in detecting non-celiac causes of villous atrophy highlight the importance of integrating histological, genetic, and microbiological evaluations into diagnostic frameworks. Technological advancements and a multidisciplinary approach are essential for overcoming the challenges of seronegative cases, ultimately enhancing diagnostic precision and patient care.

### 5.3. Differential Diagnosis

Differential diagnosis is essential in cases of villous atrophy to accurately determine the underlying cause, particularly as various conditions can present overlapping clinical and histopathological features. The differential diagnosis process should first rely on quick and inexpensive methods, namely a thorough medical history with a detailed history of pharmacotherapy. These simple tools can be helpful in the initial identification of pathogenetic factors and in guiding an in-depth diagnosis. Diagnosis of celiac disease focuses mainly on serological markers such as tTG-IgA and EMA, both of which are highly reliable in identifying gluten-induced damage. However, the absence of these markers in SNEs underscores the need for complementary diagnostic tools, such as clinical histories, histological examinations, and dietary response evaluations. Patients presenting with IgA deficiency, an often-overlooked factor affecting 2–3% of individuals, require alternative tests like deamidated gliadin peptide IgG (DGP-IgG) to circumvent the limitations of standard assays. While these options enhance diagnostic reliability, a precise evaluation of testing protocols is critical to ensure accurate results across diverse patient populations [17,94].

Drug-induced enteropathies, including those attributed to olmesartan, present histological similarities to CD, yet lack specific serological markers. Thorough patient histories, particularly regarding recent drug usage, are paramount in these cases. A hallmark of drug-induced enteropathy diagnosis is the resolution of symptoms and histological improvement upon discontinuation of the offending agent. This approach effectively distinguishes medication-related causes from CD. Olmesartan-associated villous atrophy exemplifies this challenge, as its presentation closely mimics gluten-induced damage, necessitating detailed clinical investigations to avoid misdiagnosis and inappropriate gluten-free diet interventions [12,39].

Infectious agents, such as *Giardia lamblia* and *Cryptosporidium*, often complicate the diagnostic process due to their ability to induce villous atrophy and share symptomatic overlap with CD. Pathogen identification through stool analysis PCR testing is critical to confirm an infectious etiology. Epidemiological factors, including exposure to contaminated water or regions endemic to these infections, can further guide the diagnostic process. Despite advances in diagnostic tools, underdiagnosis of parasitic infections remains a persistent issue, which underscores the need for systematic screening in patients with unexplained villous atrophy [17,25].

Histological similarities between CD and other causes, such as autoimmune enteropathy (AIE), further complicate differentiation. AIE often manifests crypt apoptosis and neutrophilic infiltration, which are uncommon in gluten-induced lesions. The pathologist’s expertise is crucial in identifying these distinct histologic patterns, underscoring the value of multidisciplinary collaboration in clinical diagnosis. Additionally, AIE may exhibit concurrent autoimmune conditions, such as type 1 diabetes or autoimmune thyroiditis, further reinforcing the need for comprehensive immunological evaluations [17,25,95].

Seronegative CD (SNCD) presents a unique diagnostic challenge, as it accounts for approximately 9% of villous atrophy cases but lacks the serological markers typically associated with CD. A definitive SNCD diagnosis hinges on demonstrating histological improvement and symptom resolution following a gluten-free diet. Given the lack of a robust serological framework, delayed diagnoses remain a common problem, resulting in prolongation of the disease process with its subsequent implications of malabsorption and nutritional deficiencies. Delayed diagnoses pose a particular threat to children and adolescents. In this case, late diagnosis can contribute to impaired growth and development of young organisms. Early suspicion and dietary trials for symptomatic individuals can optimize outcomes, but this approach requires careful exclusion of alternative etiologies through histological and clinical evaluation [17,25].

Conditions such as common variable immunodeficiency (CVID) further confound the diagnostic process due to their frequent lack of serological markers and overlapping histopathological features with other causes of villous atrophy. Immunoglobulin profiling is instrumental for identifying immune dysfunction in such cases. The early implementation of immunoglobulin replacement therapy often yields significant gastrointestinal symptom relief and reduces villous damage, highlighting the critical role of prompt recognition and intervention. However, the diagnostic complexity of CVID-associated enteropathy necessitates a nuanced approach that incorporates both immunological and histological evaluations [5].

The discovery of genetic mutations, such as CTLA4 haploinsufficiency, has provided new avenues for diagnosing refractory cases of villous atrophy. These monogenic immune disorders can mimic seronegative enteropathies or SNCD but display distinct immunopathological characteristics. Comprehensive genetic testing is increasingly vital in differentiating these conditions, particularly in cases resistant to standard treatments. By identifying the underlying molecular pathways, genetic testing enables more targeted therapeutic strategies and improves diagnostic specificity [17,25,93].

Non-celiac causes of villous atrophy often require careful reassessment when dietary interventions fail to yield results. For instance, non-response to a gluten-free diet should prompt investigations into alternative etiologies, such as autoimmune enteropathies or drug-induced enteropathies. This diagnostic step is crucial to avoid misdiagnoses and inappropriate treatments, which can exacerbate patient morbidity. Targeted histological reevaluation and collaboration with immunology specialists often clarify ambiguous cases, ensuring accurate and timely management [17].

Autoimmune enteropathy and CVID further highlight the diagnostic challenge posed by immunological disorders. Features such as crypt apoptosis and inflammatory infiltrates distinguish AIE from CD, whereas CVID-associated enteropathy often presents with recurrent infections and systemic immune dysfunction rather than gluten sensitivity. Immunoglobulin profiling and evidence of chronic viral infections, such as norovirus, are critical components of a diagnostic framework that separates these conditions from one another and CD [17,25].

The coexistence of autoimmune enteropathy and systemic autoimmune diseases necessitates thorough screening for associated conditions, such as type 1 diabetes or autoimmune thyroiditis. Identifying these concurrent conditions not only aids in differential diagnosis but also offers a more holistic understanding of the patient’s immunological profile. This integrative approach ensures that therapeutic interventions are appropriately targeted and effective in managing the underlying causes of villous atrophy [17].

Infectious agents capable of causing villous atrophy, such as *Tropheryma whipplei* and *Mycobacterium tuberculosis*, pose additional diagnostic complexities. Whipple’s disease and intestinal tuberculosis often present histological patterns, including villous atrophy and chronic inflammation, that mimic CD. Positive staining for *Tropheryma whipplei* or granulomas in biopsy samples and microbiological testing like PCR or Ziehl–Neelsen staining is indispensable for distinguishing these infections from gluten-mediated damage [17,25].

Chronic viral infections, such as norovirus in CVID patients, exacerbate villous atrophy and complicate differentiation from CD. Virological assessments and targeted antiviral therapies are critical in confirming and managing these infection-related cases. Despite advancements in diagnostics, the persistent challenge of recognizing viral contributions to villous damage underscores the need for enhanced molecular diagnostic approaches to improve patient outcomes [18].

Tropical sprue exemplifies the interplay between metabolic dysfunction and environmental influences in villous atrophy. This condition, closely linked to bacterial overgrowth and malnutrition, often resolves with tetracycline and folate supplementation. Differentiating tropical sprue from CD requires attention to geographical and dietary histories and clinical responses to specific interventions. While antibiotic and nutritional therapies have demonstrated effectiveness, incomplete reactions in some cases suggest the need for individualized metabolic profiling to inform treatment strategies [25].

Drug-induced enteropathies, including those caused by mycophenolate mofetil, highlight the importance of reviewing patient medication histories when approaching villous atrophy diagnoses. These conditions often involve mechanisms such as glucose malabsorption and mucosal inflammation, complicating the clinical picture. Integrating histological findings with detailed drug usage histories ensures the accurate exclusion of CD and other immunologically mediated causes. Emerging studies emphasize the necessity of tailored therapeutic approaches in addressing these medication-related challenges [5,17].

Histological features such as crypt hyperplasia, intraepithelial lymphocytosis, and villous blunting are standard to olmesartan-induced enteropathy and CD, which should be considered in the differential diagnosis process. The absence of a gluten-specific immune response in serologic tests is a helpful criterion in distinguishing between these conditions. Recent research has explored the role of individualized drug regimens and alternative therapies to mitigate mucosal damage, although further studies are needed to optimize patient care [25].

Genetic and molecular diagnostic tool advancements offer new opportunities to identify conditions resembling CD. Monogenic immune disorders, including those associated with CTLA4 mutations, demonstrate distinct immunological and histological profiles, enhancing diagnostic accuracy in cases resistant to standard interventions. By integrating these tools into clinical practice, clinicians can refine differential diagnoses and develop more personalized treatment strategies [17,24].

Gene–environment interactions play a significant role in certain conditions, such as tropical sprue, where environmental factors interact with genetic predispositions to induce villous atrophy. This highlights the need to explore molecular and ecological data to advance diagnostic methodologies. Incorporating these insights could provide a more comprehensive understanding of complex etiologies and tailor management strategies accordingly [5].

Emerging demographic data, such as the observed higher prevalence of villous atrophy in women and the median biopsy age of 28 years, underscore the nuanced interplay of genetic, environmental, and demographic factors in its onset. These findings call for a more holistic diagnostic approach that accounts for diverse patient characteristics to enhance diagnostic precision and therapeutic success [96].

In conclusion, a comprehensive and multidisciplinary framework is crucial for addressing the diverse causes of villous atrophy; many closely mimic CD in their clinical and histological presentation. Combining serologic, histologic, microbiologic, and molecular analyses with a detailed patient history provides an accurate differential diagnosis and allows optimization of treatment outcomes.

## 6. Conclusions

This publication’s comprehensive analysis of NCVA successfully addressed the goal of clarifying its pathophysiology, clinical manifestations, and diagnostic approaches, while differentiating it from CD. By focusing on various etiological categories—including autoimmune conditions, immune disorders, infectious agents, drug-induced enteropathies, and environmental or metabolic factors—this study highlights the complexity of NCVA as a distinct and multifaceted clinical entity. This publication provides a deeper understanding of NCVA and its implications for diagnostic accuracy and treatment strategies in clinical practice through detailed analyses of these mechanisms.

The findings underscore the complex role of villus atrophy in disrupting the primary function of nutrient absorption in the small intestine, leading to significant malabsorption and systemic complications secondary to nutritional deficiencies. It has been shown that this disorder can result from different and overlapping pathophysiological processes. Autoimmune conditions, such as autoimmune enteropathy, involve severe immune dysregulation characterized by crypt apoptosis, goblet cell depletion, and systemic autoimmunity. Immunodeficiency disorders, particularly common variable immunodeficiency (CVID), emerged as notable contributors to NCVA through chronic infections, sustained inflammation, and impaired immunoglobulin production. Infectious agents, including *Giardia lamblia*, *Cryptosporidium*, and *Tropheryma whipplei*, demonstrated how pathogens incite villous damage by epithelial apoptosis and chronic inflammation. Drug-induced enteropathies have also been described in great detail, with particular emphasis on drugs such as olmesartan and mycophenolate mofetil, which disrupt intestinal architecture through immune and metabolic pathways. Additionally, environmental conditions such as tropical sprue illuminated the interaction of bacterial overgrowth, nutritional deficiencies, and sanitation challenges that contribute to NCVA’s pathogenesis. Collectively, these findings emphasize the necessity of distinguishing NCVA from CD, given their overlapping clinical and histological presentations but differing underlying mechanisms and treatment approaches.

The paper also discusses the diagnostic complexities associated with NCVA, presenting a detailed fra.mework of histopathological, serological, genetic, and molecular tools available for differentiation and accurate identification. Histopathology, as the cornerstone of diagnosis, was shown to identify key features such as degrees of villous atrophy, crypt hyperplasia, and intraepithelial lymphocytosis, which, when evaluated alongside histological patterns like crypt apoptosis in autoimmune enteropathy or granulomas in Crohn’s disease, can refine differential diagnoses. Serological testing, while highly effective in identifying gluten-mediated conditions via tTG-IgA and EMA, was found to have limited applicability in seronegative enteropathies and conditions unrelated to gluten. Alternative markers, such as IgG (DGP-IgG), were highlighted for patients with IgA deficiency, though the diagnostic limitations and need for integrated clinical evaluations remain evident. Molecular diagnostics, including genetic testing for mutations like CTLA4 haploinsufficiency and IL-10 receptor deficiencies, emerged as invaluable tools in identifying monogenic immune disorders underlying certain cases of villous atrophy, particularly in refractory patients or those with overlapping clinical features. These advancements, integrated with traditional methodologies, offer the potential for improved diagnostic specificity and personalized therapeutic interventions.

The guiding research question—how NCVA differs from CD in its pathophysiology, clinical presentation, and management—was systematically addressed throughout the work. By detailing the immune, inflammatory, infectious, and metabolic mechanisms driving NCVA, this study highlighted the diverse pathways that distinguish it from CD and emphasized the necessity of tailored management strategies. The literature analysis conducted as part of this study demonstrated that at times when CD requires strict adherence to a gluten-free diet, NCVA often requires disease-specific approaches, such as immunosuppressive therapy in autoimmune enteropathy, immunoglobulin transfusion in CVID, targeted antimicrobial treatment for infections, and drug cessation along with supportive care for drug-induced cases. The study thus achieved its goal of identifying NCVA as a unique clinical challenge, reinforcing the importance of accurate diagnoses for the therapeutic process and improving patient outcomes.

The results of this work align with but also expand upon existing literature on NCVA. Previous studies have characterized autoimmune enteropathy and CVID as significant contributors to NCVA; however, the detailed exploration of their histological patterns, associated systemic conditions, and treatment responses presented here offers new insights into their management. Similarly, while drug-induced villous atrophy is well documented, the focus of this literature review on olmesartan and mycophenolate mofetil provides a better understanding of their distinct pathogenetic mechanisms and clinical manifestations. Exploring tropical sprue, an environmentally influenced condition, emphasizes the need for public health interventions to complement individual treatment strategies. Despite these contributions, the work also reveals gaps in understanding, such as the molecular underpinnings of idiopathic villous atrophy and the gene–environment interactions influencing susceptibility to NCVA in conditions like tropical sprue. Addressing these areas in future research could further clarify the etiology and improve the management of NCVA.

The analysis of the literature, performed as part of this article, is not without limitations. The reliance on secondary data from existing literature introduces potential biases and fragmentation, given the varied quality and focus of available research. Moreover, topics such as the precise molecular mechanisms underlying drug-induced enteropathies and the role of environmental pollutants in NCVA remain incompletely understood due to the lack of large-scale integrative studies. Additionally, while recent advancements in molecular diagnostics were discussed, their limited accessibility and thus application in clinical practice restrict their current impact on patient care. The absence of extensive population-based studies on NCVA prevalence also limits the generalizability of these findings and highlights the need for further research into underrepresented patient populations.

This work calls for robust, multicenter clinical studies to investigate the unresolved questions surrounding NCVA. Research into drug-induced enteropathies could benefit from a focus on pharmacogenomics, exploring genetic factors that predispose individuals to villous damage and informing safer prescribing practices. Additionally, molecular and genetic studies should prioritize refining the classification of monogenic immune disorders linked to NCVA, enabling the development of targeted therapies. Public health interventions to improve sanitation and nutrition in regions affected by tropical sprue are essential to reduce disease burden. New diagnostic frameworks leveraging artificial intelligence could standardize biopsy evaluations, reducing observer variability and enhancing diagnostic accuracy. Finally, future research should explore the epigenetic and environmental contributions to NCVA, particularly in conditions with complex gene–environment interactions, to advance precision medicine approaches and optimize patient care.

Reflecting on the broader implications of this work, it underscores the critical need for heightened clinical awareness of NCVA as an underrecognized and diagnostically challenging condition. Advances in diagnostic and therapeutic strategies, as delineated here, hold the potential to significantly improve patient outcomes by enabling earlier detection and more effective, individualized treatments. The collaboration required in gastroenterology, immunology, genetics, and public health underscores the importance of an interdisciplinary approach to better understand and treat NCVA. By addressing the complexity of pathogenesis and integrating various diagnostic tools, this study significantly contributes to the growing knowledge of gastrointestinal disorders. It paves the way for future research to fill remaining gaps in understanding.

## Figures and Tables

**Table 1:** Etiology and characteristics of specific types of NCVA [3,9,18,19,20,22].

Cause	Etiology	Type	Clinical Manifestation	Histological Findings
Primary Causes	Autoimmune Conditions and Disorders	Autoimmune Enteropathy (AIE)	Persistent diarrhea Malabsorption Weight loss	Villous atrophy Minimal intraepithelial lymphocytosis Apoptotic bodies in crypts Absence of cup cells or Paneth cells
		Type 1 Diabetes Mellitus (T1DM)	Malabsorption Glycemic instability Suboptimal diabetes management poor glycemic control	Lack of specific histological findings
		Autoimmune Thyroiditis (Hashimoto’s Thyroiditis; Grave’s Disease)	Malabsorption Osteoporosis Anemia Dysregulated systemic metabolism and hyper/hypothyreosis symptoms	Lack of specific histological findings
		Systemic Lupus Erythematosus (SLE)	Diarrhea Malabsorption Mimicry to other villous atrophy causes Serological markers associated with SLE (anti-dsDNA; anti-SM antibodies)	Lack of specific histological findings
		Autoimmune Polyglandular Syndromes (APS)	Adrenal insufficiency, T1DM, autoimmune thyroiditis in APS type II Nutrient malabsorption	Lack of specific histological findings
	Immunodeficiency Disorders	Common Variable Immunodeficiency Disease (CVID)	Diarrhea Weight loss Malabsorption syndrome Insufficient immunoglobulin production Recurrent infections	Lack of specific histological findings
		HIV/AIDS	Malabsorption Osteomalacia Anemia Hypocholesterolemia HIV/AIDS-associated infections	Enterocyte injury Partial villous atrophy Crypt hyperplasia
	Infectious Etiology	*Giardia lamblia*	Malabsorption Diarrhea Weight loss Nutritional deficiencies	Mucosal inflammation Disruption of tight junctions in epithelial cells Crypt hypertrophy with associated atrophic changes
		*Cryptosporidium*	Very severe malabsorption	Epithelial cell death Flattening of the villi
		*Noroviruses*	Treatment-resistant diarrhea Impaired nutrient absorption Prolonged malnutrition	Permanent villous damage
		*Mycobacterium tuberculosis*	Impaired absorption	Granulomatous inflammation Villous destruction
		*Tropheryma whipplei* (Whipple’s disease)	Malabsorption	Lamina propria infiltration Macrophage accumulation Flattening of the villi
		Tropical Splenomegaly Syndrome (TSS)	Bacterial dysbiosis Nutritional deficiencies Chronic diarrhea Weight loss Malabsorption Splenomegaly	Villous atrophy
Secondary and Iatrogenic Causes	Drug-Induced Enteropathy	Non-Steroidal Anti-Inflammatory Drugs (NSAIDs)	Lack of specific clinical findings	Chronic inflammation Mucosal damage Villous atrophy
		Angiotensin Receptor Blockers (ex. Olmesartan)	Severe diarrhea Weight loss Dehydration Organ failure	Villous atrophy Lymphocytic infiltration Shortened intestinal villi Overlapping with celiac disease
		Mycophenolate Mofetil (MMF)	Malabsorption Abnormal glucose absorption and chloride loss	Villous atrophy
	Environmental Factors	Alcohol	Impairment of nutrient absorption Diarrhea Weight loss	Reversible villous atrophy
		Bacterial Toxins (ex. tropical sprue)	Bacterial dysbiosis Nutrient malabsorption Diarrhea	Villous atrophy
		Dietary toxins/food additives/environmental pollutants (heavy metals, pesticides)	Lack of specific clinical findings	Villous atrophy Microbiota disruption Low-grade inflammation
	Metabolic Disorders	Congenital Enteropathy	Decreased glucose and sodium absorption Severe malnutrition Dehydration	Hypoplastic villous atrophy Absence of a brush border Absence of inflammation
	Genetic Disorders	Hereditary alpha-tryptasemia	Chronic abdominal pain Diarrhea/constipation, Gastroesophageal reflux, Bloating Irritable bowel syndrome-like symptoms Postural orthostatic tachycardia syndrome (POTS)	Villous atrophy Increased number of mast cells

## Data Availability

No new data were created or analyzed in this study. Data sharing is not applicable to this article.

## Abbreviations

The following abbreviations are used in manuscript:

AI	Artificial Intelligence
AIDS	Acquired Immunodeficiency Syndrome
AIE	Autoimmune Enteropathy
APS	Autoimmune Polyglandular Syndromes
ARBs	Angiotensin Receptor Blockers
CD	Celiac Disease
CCND2	Cyclin D2
CTLA4	Cytotoxic T-Lymphocyte Associated Protein 4
CVID	Common Variable Immunodeficiency
CVIDs	Immunodeficiency-related enteropathies
DGP-IgG	Deamidated Gliadin Peptide IgG
DOAJ	Directory of Open Access Journals
DVL2	Disheveled 2 Protein
EMA	Anti-Endomysial Antibodies
EATL	Enteropathy-Associated T-cell Lymphoma
HIV	Human Immunodeficiency Virus
HLA	Human Leukocyte Antigen
IFN-γ	Interferon-Gamma
Igs	Immunoglobulins
IL	Interleukin
IL10RA	Interleukin 10 Receptor Subunit Alpha
IVA	Idiopathic Villous Atrophy
MDPI	Multidisciplinary Digital Publishing Institute
MMF	Mycophenolate Mofetil
NCVA	Non-Celiac Villous Atrophy
NCEs	Non-Celiac Enteropathies
NSAIDs	Non-Steroidal Anti-Inflammatory Drugs
PCR	Polymerase Chain Reaction
ROS	Reactive Oxygen Species
SLE	Systemic Lupus Erythematosus
SNCD	Seronegative Celiac Disease
SNEs	Seronegative Enteropathies
SNVA	Seronegative Villous Atrophy
SOT	Solid Organ Transplant
T1DM	Type 1 Diabetes Mellitus
TLA	Three-Letter Acronym
TNF-α	Tumor Necrosis Factor-Alpha
TPN	Total Parenteral Nutrition
tTG-IgA	Tissue Transglutaminase IgA
VA	Villous Atrophy
